# Bioformulation of *Bacillus proteolyticus* MITWPUB1 and its biosurfactant to control the growth of phytopathogen *Sclerotium rolfsii* for the crop *Brassica juncea* var local, as a sustainable approach

**DOI:** 10.3389/fbioe.2024.1362679

**Published:** 2024-04-19

**Authors:** Humaira Mukadam, Shikha V. Gaikwad, Nithya N. Kutty, Vikrant D. Gaikwad

**Affiliations:** ^1^ Department of Biosciences and Technology, School of Science and Environment Studies, Faculty of Science and Health Science, Dr. Vishwanath Karad MIT World Peace University, Pune, Maharashtra, India; ^2^ Department of Chemical Engineering, School of Engineering and Technology, Faculty of Engineering, Dr. Vishwanath Karad MIT World Peace University, Pune, Maharashtra, India

**Keywords:** *Bacillus proteolyticus*, biosurfactant, plant growth-promoting traits, bioformulation, *Brassica junce*a var L, *Sclerotium rolfsii*

## Abstract

Bacillus proteolyticus MITWPUB1 is a potential producer of biosurfactants (BSs), and the organism is also found to be a producer of plant growth promoting traits, such as hydrogen cyanide and indole acetic acid (IAA), and a solubilizer of phosphate. The BSs were reportedly a blend of two classes, namely glycolipids and lipopeptides, as found by thin layer chromatography and Fourier-transform infrared spectroscopy analysis. Furthermore, semi-targeted metabolite profiling via liquid chromatography mass spectroscopy revealed the presence of phospholipids, lipopeptides, polyamines, IAA derivatives, and carotenoids. The BS showed dose-dependent antagonistic activity against *Sclerotium rolfsii*; scanning electron microscopy showed the effects of the BS on *S. rolfsii* in terms of mycelial deformations and reduced branching patterns. *In vitro* studies showed that the application of *B. proteolyticus* MITWPUB1 and its biosurfactant to seeds of *Brassica juncea* var local enhanced the seed germination rate. However, sawdust-carrier-based bioformulation with *B. proteolyticus* MITWPUB1 and its BS showed increased growth parameters for *B. juncea* var L. This study highlights a unique bioformulation combination that controls the growth of the phytopathogen *S. rolfsii* and enhances the plant growth of *B. juncea* var L. *Bacillus proteolyticus* MITWPUB1 was also shown for the first time to be a prominent BS producer with the ability to control the growth of the phytopathogen *S. rolfsii*.

## 1 Introduction

Plants are severely affected by microbial pathogens that reduce their productivity. According to forecasts by the Food and Agricultural Organization (FAO, 2020), crop production is expected to increase by 70% by 2026, when the global population is expected to be approximately nine billion. Satisfying the food demands of the growing population will therefore be a huge challenge worldwide and a vital requirement. Significant doses of agrochemicals have been applied in fields for decades to enhance crop productivity. High concentrations of chemicals can result in repercussions that disturb the equilibrium status of the environment and affect the health of the macro-microorganisms (Brauer et al., 2019). Water shortage, soil deterioration, nutrient limitations, and climate change can further exacerbate these conditions (Mishra et al., 2020; Jamal et al., 2023). Therefore, establishing sustainable and environmentally friendly farming techniques is crucial and requires thoughtful consideration.

The plant growth promoting (PGP) traits shown by bacteria for enhancing the productivity of plants and soil have been well-documented in the literature for a long time. These traits help plants by producing phytohormones, growth regulators (Pantoja-Guerra et al., 2023), and surface-active chemicals like biosurfactants (BSs) that enhance the absorption of nutrients (Egamberdieva et al., 2019). Biosurfactants are classified according to their molecular weights, chemical structures, and microbiological sources into glycolipids, lipopeptides, phospholipids, fatty acids, and polymeric macromolecules (Santos et al., 2016); they are biodegradable, biocompatible, and stable under various environmental conditions, which highlights their efficiency for use as promising molecules in green technologies. BSs promote the motility of bacteria, thereby enhancing their root colonization ability in plants. Furthermore, they enhance the nutrient uptake of plants as well as their capability for disease resistance and tolerance to abiotic stressors (Primo et al., 2015). The amphiphilic nature of a BS offers excellent ability to lower the interfacial and surface tensions that play effective roles in bioremediation of xenobiotic compounds (Jamal et al., 2023). Microbes with PGP features and the capacity to produce biostimulants have the potential to be employed as ecologically friendly options for boosting agricultural yield and combating problems sustainably (Adedayo and Babalola, 2023). In the present study, soil samples contaminated with hydrocarbons were used to isolate bacteria so as to access their PGP efficacies.

*Brassica juncea* is a well-known oilseed crop in India and is grown all over the world (Shekhawat et al., 2012). Plant oils have higher percentages of unsaturated fatty acids than other edible oils (Baux et al., 2008), making them more acceptable for consumption. However, mustard production in India is limited by several biotic and abiotic variables (Dhaliwal et al., 2021). *B. juncea* is an important crop that suffers from stem rot disease caused by the phytopathogen *Sclerotium rolfsii.* This disease is identified by the appearance of white or brownish lesions on the stem, which eventually cause the plant to wilt and die. The phytopathogen survives because of its sclerotia and persists in soil for a longer period. To address this problem, bacteria with PGP traits have been used as bioinoculants as they are environmentally friendly alternatives that increase plant health, productivity, soil nutritional status, and xenobiotic chemical degradation rates while protecting the plants from abiotic challenges and reducing dependency on synthetic chemicals. The present work elucidates the isolation of bacteria with PGP traits for producing a BS. Furthermore, the potential of the bacteria and its metabolite BS was evaluated for inhibiting the growth of the phytopathogen *S. rolfsii* and enhancing the *B. juncea* var local oilseed crop growth under *in vitro* conditions in bioformulation studies.

## 2 Materials and methodology

### 2.1 Media

Potato dextrose broth (PDB), nutrient broth (NB), Luria agar (LA), potato dextrose agar (PDA), and minimal salt medium (MSM) were purchased from HiMedia Laboratories Pvt. Ltd.

### 2.2 Preparation of bacterial isolates and procurement of fungal culture

Soil samples used in this study were collected from hydrocarbon-contaminated regions of Pune (18.5204° N, 73.8567° E), Maharashtra, India, in a presterilized vial and maintained at 4°C for bacterial isolation. After serial dilutions, the soil sample was spread on the MSM and incubated at 37°C for 72 h. Morphologically diverse bacterial colonies were then chosen and affirmed on LA medium at 4°C for further experiments. *Sclerotium rolfsii* fungal culture was procured from Agarkar Research Institute (ARI) in Pune and maintained at 4°C on PDA for further use.

### 2.3 Screening of bacterial isolates for PGP traits

#### 2.3.1 Indole acetic acid production assay

To determine IAA ability, the Salkowski colorimetric assay was performed according to Lebarzi et al. (2020). The concentration of IAA produced by the bacterial isolate was determined using the standard curve of IAA.

#### 2.3.2 Hydrogen cyanide production assay

HCN producing ability was verified according to Lorck, (1948), where a color change from yellow to orange or brown indicates a positive result.

#### 2.3.3 Solubilization of phosphate

The solubilization activities of the bacterial isolates were assessed in a Pikovskaya (PVK) agar plate; a 24-h-old bacterial culture was spot inoculated and incubated at 37°C for 120 h on a PVK plate. A clean halo zone around the spotted colony indicates a positive result (Babiye, 2022).

### 2.4 Screening of bacterial isolates for BS production

Each bacterial isolate was grown in the MSM supplemented with 1% (v/v) vegetable oil as the sole source of carbon and incubated at 37°C and 150 rpm for 240 h. Upon incubation completion, the broth was centrifuged at 8,400 rpm for 30 min at 4°C to obtain the cell-free supernatant. The BS production abilities of the bacterial isolates were evaluated via emulsification index measurement and drop collapse assay (Shah et al., 2016).

#### 2.4.1 Drop collapse assay

The supernatant from an overnight-grown bacterial culture was placed on a glass slide along with crude oil in the ratio of 2:1 (v/v) and maintained at room temperature for 2–3 min under undisturbed conditions. Later, its time of collapse was observed. Sodium dodecyl sulfate (10%, w/v) was used as the positive control, and distilled water was used as the negative control (Zargar et al., 2022).

#### 2.4.2 Emulsification index

Crude oil and an overnight-grown culture of a bacterial cell-free supernatant were mixed in the ratio of 1:1 (v/v). This mixture is vortexed vigorously for 1 min and left undisturbed for 18 h at room temperature. Then, the emulsification index is determined by the methodology described in Zargar et al. (2022) using sodium dodecyl sulfate (10%, w/v) as the positive and distilled water as the negative controls.

### 2.5 Kinetics of potential BS production by bacterial isolate

MSM (100 mL) amended with 1% (v/v) vegetable oil at 7.0 pH was maintained; then, this medium was inoculated with 2% (v/v) bacterial cell suspension (inoculum density, colony forming unit; CFU per mL 10^7^, OD 6.8 at 600 nm), incubated at 37°C (Durval et al., 2018; Hisham et al., 2019), and agitated at 150 rpm for 240 h. After incubation completion, the broth was centrifuged at 8,400 rpm for 30 min at room temperature, and the pellets were dried out in a hot-air oven at 50°C (Sen et al., 2017) to be considered cell biomass. Later, the cell-free broth was centrifuged at 8,400 rpm and 4°C before being used to extract the BS. Then, the pH was reduced to 2 by addition of 6N HCl to the cell-free broth and left overnight at 4°C. Chloroform and methanol were added in the ratio of 1:1 (v/v) to the cell-free broth and stirred for 10 min. The upper phase was collected and evaporated to obtain the BS (Zargar et al., 2022). The yields of cell biomass and BS were calculated as per the methodology described by Sen et al. (2017).

#### 2.5.1 Emulsification stability of BS at various pH, temperature, and salinity conditions

The stability of the BS was evaluated at different temperatures (4°C, 27°C, 37°C, and 50°C) and pH values (3, 5, 7, 9, and 11) as per the methodology described by Sharma et al. (2015); the salinity conditions were evaluated as per Fardami et al. (2022). The salinity was maintained by the addition of sodium chloride at 1%, 2%, 3%, and 4% (w/v) to the cell-free broth.

### 2.6 Characterization of the BS

#### 2.6.1 Thin layer chromatography

Preliminary characterization of the BS was done by thin layer chromatography (TLC). The BS was redissolved in deionized water, spotted onto a silica gel plate (Merck DC, Silica gel 60 F_254_), and assessed in the presence of methanol as a mobile phase. Post-chromatography, the plates were air dried and sprayed with the respective reagents before incubation in a hot-air oven at 110°C for 10 min to observe the color changes under ultraviolet and visible light. The presence of peptides and amino acids was detected using the ninhydrin test, presence of lipids was determined by the iodine vapor test, and presence of sugars was detected by the Anthrone test (Zargar et al., 2022).

#### 2.6.2 Fourier-transform infrared (FTIR) spectroscopy

To identify the functional groups and bonding patterns in the crude BS, FTIR spectroscopy was performed. According to Hannah et al. (2020), the FTIR spectrum between the wavenumber values of 400 and 4,000 cm^-1^ was recorded using the Bruker Alpha II Platinum instrument in the attenuated total reflectance (ATR) mode.

#### 2.6.3 Untargeted metabolite profiling using liquid chromatography–mass spectroscopy (LC-MS)

LC-MS was performed on the extracted bacterial metabolite at the NCL Venture Centre, Pune, Maharashtra, India. Using the Agilent XDB-C18 column (3 × 150 mm, 3.5 µ), separation was performed at 40°C. Sample gradient elution was carried out using 0.1% (v/v) formic acid in water (A) and 0.1% (v/v) formic acid in acetonitrile (B) at a mobile flow rate of 0.3 mL per min. The mobile phase gradient used was 0 min at 95% A, 2 min at 95% A, 25 min at 5% A, 28.1 min at 95% A, and 30 min at 95% A. The analysis was carried out using the Agilent Q-ToF G6540B device connected to the Agilent 1,260 Infinity II HPLC platform. Detection was performed using a mass-ion-source dual AJS ESI for positive ionizations in the mass scan range of 100–1700. The compounds were identified using Agilent’s METLIN database; furthermore, a few compounds were identified by comparing the predicted molecular weights, molecular formulas, and mass spectra with databases such as ChemSpider and PubChem. The LC-MS data of the metabolite were submitted to MetaboLights platform as per the steps described by Yurekten et al. (2024).

### 2.7 Impact of BS on the growth of phytopathogen *S. rolfsii*

#### 2.7.1 Evaluating the antifungal activity of BS against phytopathogen *S. rolfsii* using the poison food technique

The fungus *S. rolfsii* was procured from the Agarkar Research Institute in Pune, Maharashtra, India, and grown on PDA medium at 28°C for further study. The antifungal activity of the BS was checked by the poison food technique, where 6-mm wells were prepared at four corners of the plate. The BS at two concentrations, i.e., 10 and 30 mg per mL, was poured into each well. A 6-mm plug of *S. rolfsii* from a 72-h-old plate was excised and placed in the middle of the fresh PDA plate. The antibiotic cycloheximide (5 mg per mL) was used as a positive control, and distilled water was used as the negative control. Test samples (100 µL) with positive and negative controls were added to the plate. The plate was then incubated for 72 h at 28°C; the inhibition zone (in mm) indicating the effectiveness of the compound for the growth of *S. rolfsii* was observed around each well, and the growth inhibition rate for *S. rolfsii* was calculated.

#### 2.7.2 Dry weight of fungal cell biomass

PDB (20 mL) was inoculated with 1 mL of 10^5^ spores per mL suspension of *S. rolfsii*. Two different concentrations of the BS (10 and 30 mg per mL) were added to this medium and incubated separately at 27°C for 120 h at 100 rpm. After incubation, each medium was centrifuged for 30 min at 10,000 rpm and room temperature to obtain pellets of *S. rolfsii*, which were dried at room temperature, weighed, and considered as the fungal cell biomass.

#### 2.7.3 Antifungal effect of BS against *S. rolfsii* using scanning electron microscopy

The pellet obtained from the flask inoculated with 30 mg per mL of the BS was used to prepare the SEM sample. The sample was first washed with phosphate buffer (pH 7.0) three times and fixed with 2.5% (v/v) glutaraldehyde before maintaining at 4°C for 16 h. Later, the sample was washed serially with different concentrations of ethanol (30, 50, 70, 80, 90, 95, and 100%, v/v) for 10 min each (Borah et al., 2016) and further processed for SEM analysis (Zeiss, 6-Sigma).

### 2.8 Impact of BS on the plant growth of *B. juncea* var local

#### 2.8.1 Plant toxicity assay

BS phytotoxicity was evaluated on the seeds of *B. juncea* var local as per the methodology described by Ribeiro et al. (2020) and Mendes et al. (2021). The seeds were surface sterilized with 1% (v/v) hydrogen peroxide and further rinsed with deionized water three times. Later, the seeds were soaked in 10 and 30 mg per mL concentrations of the BS for 30 min before being placed on moist filter paper in sterilized Petri dishes for incubation at room temperature for 120 h. Seeds soaked in sterilized distilled water served as the control. The seed germination percentage was calculated according to Tiquia et al. (1996).

#### 2.8.2 Bioformulation studies and impacts on the plant growth of *B. juncea* var local and fungus *S. rolfsii in vitro*

The formulation was developed as per the method described by Khare and Arora (2021) and Mishra et al. (2020) with minor modifications. Sawdust was used as the carrier agent and amended with 5% (w/v) bacterial culture (10^8^ CFU per mL) and two concentrations of the BS, i.e., 10 and 30 mg per mL v/v with respect to the bacterial culture. This formulation was further incubated at 37°C for 360 h, with the CFU per mL being checked at regular intervals of 24 h (Paudyal et al., 2021). Distilled water without the BS was used as the control. For *in vitro* studies, approximately 250 g of the bioformulated sawdust (as per the abovementioned method), 1% (w/v) carboxymethyl cellulose (CMC), and 1.5% (w/v) calcium carbonate were added to 1 kg of soil. After addition, the formulation was maintained at room temperature for 24 h, and surface-sterilized bacterized seeds of *B. juncea* var local were sown. The seeds were germinated in a germination tray and monitored for 360 h at room temperature. As per the treatment requirements, fungal spores (10^5^ spores per mL) were added to the soil. The treatments are as follows:

1. C1: Non-bacterized seed
2. C2: Non-bacterized seed (MITWPUB1) with fungal suspension of *S. rolfsii*
3. C3: Non-bacterized seed with the biosurfactant (10 mg)
4. C4: Non-bacterized seed with the biosurfactant (30 mg)
5. P1: Bacterized seed (MITWPUB1) with the fungal suspension of *S. rolfsii*
6. P2: Bacterized seed (MITWPUB1) with the biosurfactant (10 mg)
7. P3: Bacterized seed (MITWPUB1) with the biosurfactant (30 mg)
8. P4: Bacterized seed with BS (10 mg) and fungal suspension of *S. rolfsii*
9. P5: Bacterized seed with BS (30 mg) and fungal suspension of *S. rolfsii*

#### 2.8.3 Statistical analysis

For the data analyses, one-way ANOVA and Duncan’s multiple range tests were performed using IBM SPSS Statistics version 29.0.1.0. To create the graphs, OriginPro version 2023 was used. All experiments were performed in triplicate, and the standard deviations were calculated and reflected through error bars.

### 2.9 Morphological, microscopic, and molecular identifications of the bacterial isolate

The potential bacterial isolate MITWPUB1 was identified by morphological and biochemical analyses (Garrity et al., 2005). SEM was performed on the potential isolate using the method described previously. For molecular identification, 16S rRNA sequencing was performed at the Pure Microbes Laboratory, Pune, Maharashtra, India. Furthermore, the closely related sequences were examined using BLAST of the NCBI with MEGA version 11 software, and the phylogenetic tree was constructed.

## 3 Results

### 3.1 Isolation and screening of bacteria for PGP abilities

From the hydrocarbon-contaminated soil samples, 14 morphologically different bacteria were identified and abbreviated as MITWPUB1, MITWPUB2, and so on till MITWPUB14. The bacterial isolates were tested for their PGP traits. All bacterial isolates were producers of IAA. A significant amount of IAA (Supplementary Table S1; Supplementary Figure S1) was produced by MITWPUB1 (48 µg per mL). Bacterial isolates MITWPUB1, MITWPUB2, MITWPUB3, and MITWPUB4 were also found to produce HCN. All bacterial isolates were found to be phosphate solubilizers. The maximum solubilization zone for phosphate was observed for the MITWPUB1 bacterial isolate on PKV medium (Supplementary Table S2).

### 3.2 Screening of bacterial isolates for BS production

All isolates were found to be positive for the drop collapse test, and their emulsification indexes were in the range of 4%–83%. The bacterial isolate MITWPUB1 showed an emulsification index of 83% and drop collapse within 30 s (Supplementary Table S2). Based on the PGP traits and BS production capacity, MITWPUB1 was selected as a potential strain for further studies.

### 3.3 Kinetics and stability studies of BS production

**FIGURE 1:**
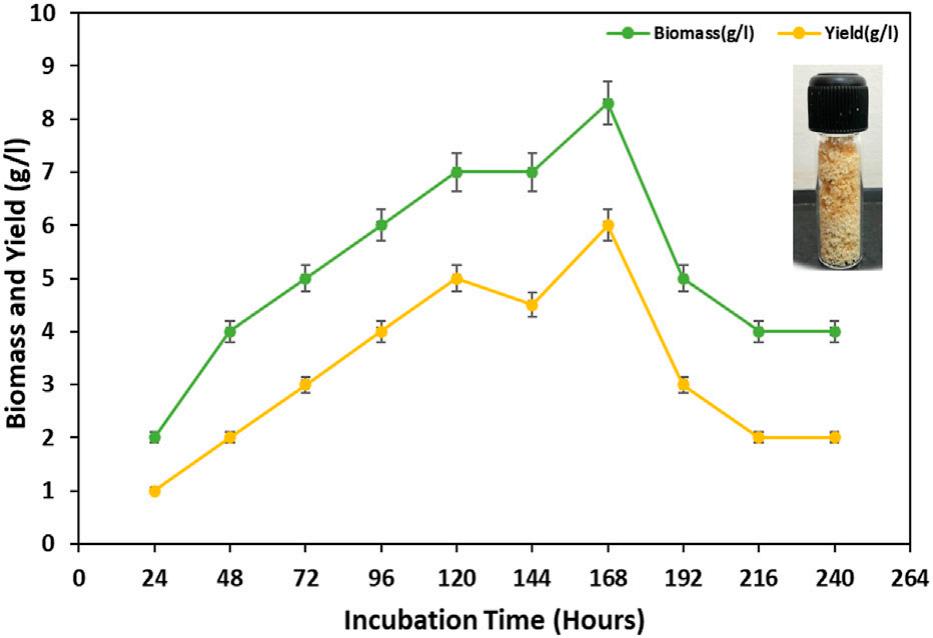
Growth kinetics profile of *Bacillus proteolyticus* MITWPUB1 with reference to its biosurfactant production as a function of time. All values are the mean ± SD of three replicates. The error bars represent standard deviations (SDs).

**FIGURE 2:**
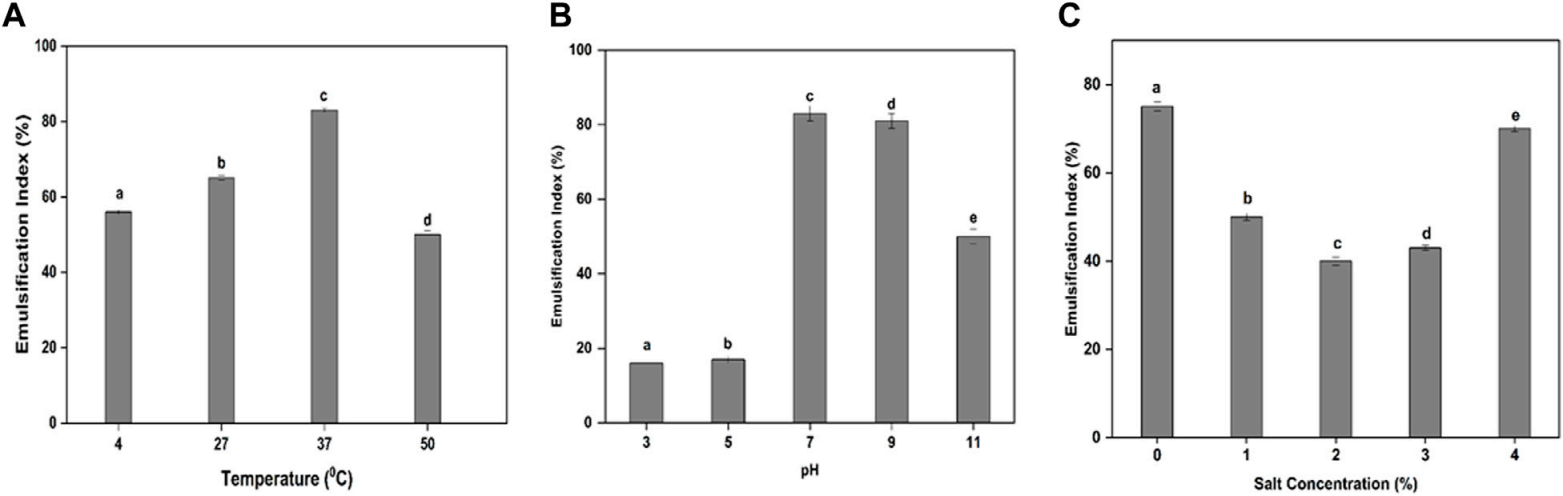
Impacts of different parameters on the emulsification index (EI): **(A)** effect of temperature (°C); **(B)** effect of pH; **(C)** effect of NaCl concentration (%, w/v). All values are the mean ± SD of three replicates. The bar graphs having the same letters are not significantly different from each other at *p* ≤ 0.05.

In this study, the maximum amounts of cell biomass and BS were obtained from the bacterial isolate MITWPUB1 after 168 h of incubation. The cell biomass was 8 g per liter, and BS obtained was 6 g per liter (Figure 1). Thereafter, a trend decline was observed till 216 h. Furthermore, a static effect was observed after 216 h of incubation till 240 h. In addition, the stability of the BS in terms of emulsification index was studied for the bacterial isolate MITWPUB1 at various pH levels, temperatures, and salinity conditions. The emulsification test showed that the BS was most stable at pH 7 and 37°C. It is interesting to note that the emulsification index was observed at even 4% salinity, which is the second highest value after that of 1% NaCl (Figures 2A–C).

### 3.4 Characterization of BS produced by MITWPUB1

#### 3.4.1 TLC

TLC was used to characterize the different moieties present in the BS, such as peptides, amino acids, sugars, and lipids. After spraying ninhydrin reagent, a purple-pink spot indicating the presence of peptides and amino acids was observed. A green spot was observed for the Anthrone test, which indicates the presence of carbohydrate moieties, and yellow-brown spots indicating the presence of iodine vapors denote the existence of lipid moieties (Supplementary Figure S2). The compound showed positive reactions for the ninhydrin, Anthrone, and iodine tests. The Rf values for the peptides/amino acids, sugars, and lipids were recorded as 0.65, 0.57, and 0.68, respectively. Based on the glycolipid and lipopeptide moieties, two classes of BSs were distinguished in the sample.

#### 3.4.2 FTIR spectroscopy

**FIGURE 3:**
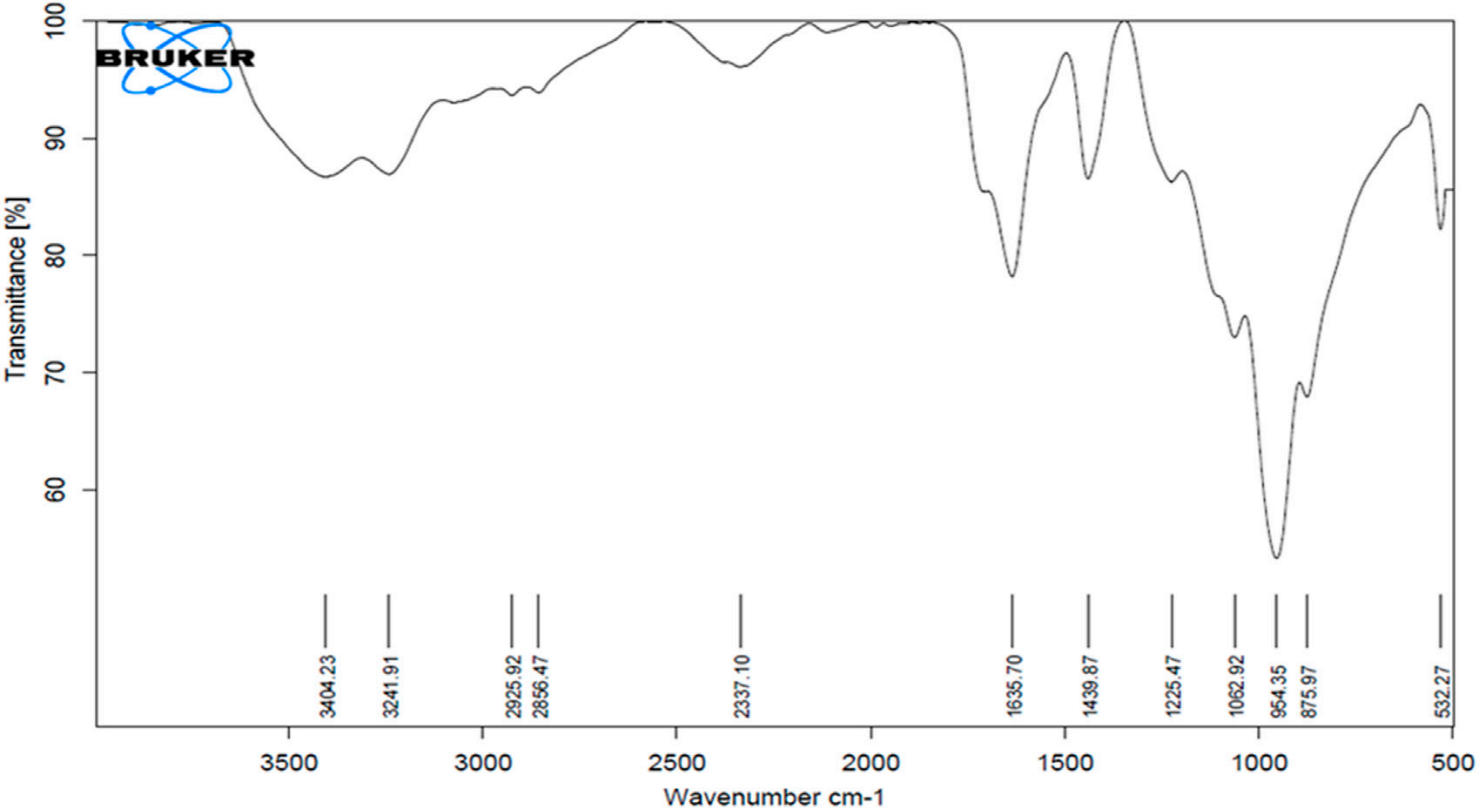
FTIR spectrum of the biosurfactant produced by *Bacillus proteolyticus* MITWPUB1.

The FTIR analysis showed absorption peaks at significant wavenumbers, confirming the presence of different moieties, such as sugars, peptides, amino acids, and lipids. The peak at 3241 cm^-1^ corresponds to the N-H stretching vibration that shows presence of the peptide moiety. The strong peak at 3404 cm^-1^ is associated with the hydroxyl (-OH) group, which denotes presence of sugar moieties or the glycerol backbone. In some fatty acid chains, an alkyne (-C-C-) group is present, which is indicated by the small peak at 2337 cm^-1^. The C=O stretching vibrations in the peptide bonds of proteins and/or ester bonds of lipids are responsible for the peak at 1635 cm^-1^. The C-H deformation vibrations in alkanes or methyl groups or the bending vibration of the carboxylate group in fatty acids is responsible for the absorbance peak at 1439 cm^-1^. The peak corresponding to C-H was determined at 954 cm^-1^. Another peak corresponding to the chloroalkane (-C-Cl) group present in several halogenated BSs was observed at 532 cm^-1^ (Figure 3).

#### 3.4.3 Untargeted metabolite fingerprinting of BS crude extract using LC-MS

Metabolite fingerprinting analysis was carried out using LC-MS analysis. Several types of phospholipids, glycerolipids, glycolipids, and sphingolipids were detected in this semi-targeted analysis. Furthermore, the presence of peptides and a few amino acid derivatives were found in the sample. Interestingly, the crude extract of the BS also indicated the presence of polyamines (spermidine, N-acetyl spermidine), carotenoids (7,8-didehydroastaxanthin), and IAA derivatives. The complete list of compounds detected in the crude extract is presented in Supplementary Table S3. The mass spectrum of each identified compound is shown in Supplementary Figure S3.

### 3.5 Impact of BS on growth of phytopathogen *S. rolfsii* under *in vitro* conditions

**FIGURE 4:**
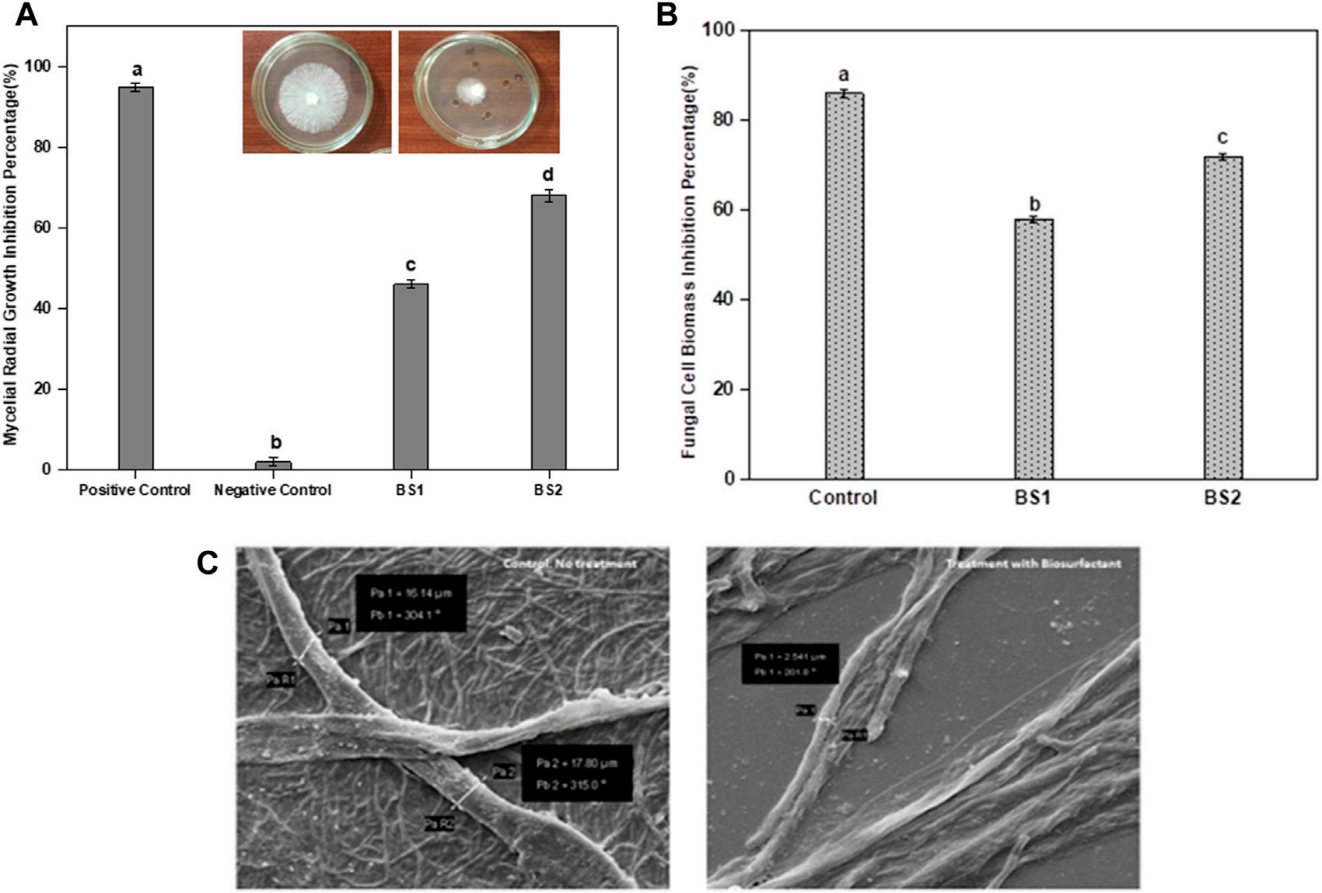
Antifungal activity of the biosurfactant in terms of percentage inhibition against **(A)** *Sclerotium rolfsii* mycelia radial growth with varying concentrations of the biosurfactant produced by *B. proteolyticus* MITWPUB1; **(B)** *S. rolfsii* cell biomass with varying concentrations of the biosurfactant produced by *B. proteolyticus* MITWPUB1. **(C)** Scanning electron microscopy of *S. rolfsii* under ×2,500 magnification. The error bars represent standard deviations (SDs). All values are the mean ± SD of three replicates. The bar graphs having the same letters are not significantly different from each other at *p* ≤ 0.05. Positive control, cycloheximide (5 mg per mL); negative control, distilled water; BS1, biosurfactant (10 mg per mL); BS2, biosurfactant (30 mg per mL). Inset in A, representative photographs of the petriplate showing growth of *S. rolfsii* after 72 h (left) and reduced growth upon exposure to the biosurfactant and positive control (right).

The inhibitory effects of the BS on the fungus *S. rolfsii* were observed at concentrations of both 10 and 30 mg per mL. Through the poison food technique, the zones of inhibition were observed. Furthermore, the antifungal activity of the BS was more pronounced at 30 mg per mL (Figure 4A). A similar pattern was observed for the dry biomass assay. The dry weight of the fungal biomass (Figure 4B) was shown to be more effectively reduced by a higher dose of the BS (30 mg per mL), which was found to be statistically significant. The effect of the BS on the fungus was clear in the SEM images. The images of *S. rolfsii* treated with BS showed significant morphological mycelial deformations, such as reduced thickness and formation of lesions in the filaments (Figure 4C), which were not observed in the controls.

### 3.6 Impact of BS on plant growth of *B. juncea* var local

#### 3.6.1 Plant toxicity assay

The impacts of the BS on the germination rates of *B. juncea* seeds were observed at two concentrations; an enhancement of seed vigor index was also observed with respect to the control. A more pronounced effect was observed when the seeds were bacterized with MITWPUB1 and pelleted with the BS of higher concentration, such as 30 mg per mL, than with the concentration of 10 mg per mL (Table 1).

**TABLE 1:** Phytotoxicity of the biosurfactant produced by *B. proteolyticus* MITWPUB1 on the seeds of *Brassica juncea* var local.

Sr. No.	Treatment	Seed germination percentage (seed vigor index)
1	Control	20
2	Test 1–MITWPUB1 bacteria treatment	46
3	Test 2–MITWPUB1 bacteria treatment + biosurfactant (10 mg per mL)	55
4	Test 3–MITWPUB1 bacteria treatment + biosurfactant (30 mg per mL)	64

#### 3.6.2 Bioformulation studies and impacts on test crop *B. juncea* var local and fungus *S. rolfsii*

**FIGURE 5:**
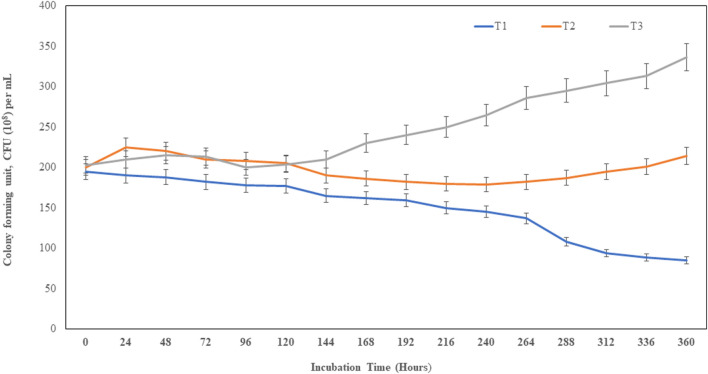
Population density (CFU per mL (10^8^) of MITWPUB1 in sawdust carrier material. The error bars represent standard deviations (SDs). T1, control (bacteria); T2, sawdust + bacteria; T3, sawdust + bacteria + biosurfactant (30 mg per mL).

The sawdust bioformulated with bacteria MITWPUB1 and its BS showed a significant increase in the CFU per mL even after 240 h up to 360 h. These results were more significant in the presence of the bacteria with its BS concentration at 30 mg per mL (Figure 5; Supplementary Figure S4). The bacteria and BS with the carrier bioformulation showed significant enhancements to the root and shoot heights of the plant (Table 2).

**TABLE 2:** Effects of inoculations of different treatments on the plant growth parameters of *B. juncea* var local*.*

Treatment	Root length (cm)	Shoot length (cm)
C1	2.5 ± 0.3^c^	9 ± 1.0^b^
C2	0.5 ± 0.2^a^	3 ± 0.5^a^
C3	3.4 ± 0.3^d^	18 ± 1.5^cd^
C4	4 ± 0.5^d^	19 ± 3.2^d^
P1	2 ± 0.2^b^	8 ± 2.5^b^
P2	4.5 ± 0.4^e^	22 ± 3.0^d^
P3	5.2 ± 0.3^e^	32 ± 3.0^f^
P4	3 ± 0.3^cd^	15 ± 2.5^bc^
P5	4.8 ± 0.5^e^	26 ± 2.6^e^

All values are given as mean ± SD. Values in the columns followed by the same letters indicate no significant differences (*p* ≤ 0.05) by Duncan’s multiple range test. Abbreviations: C1, non-bacterized seed; C2, non-bacterized seed (MITWPUB1) with fungal suspension of *S. rolfsii*; C3, non-bacterized seed with biosurfactant (10 mg); C4, non-bacterized seed with biosurfactant (30 mg); P1, bacterized seed (MITWPUB1) with fungal suspension of *S. rolfsii*; P2, bacterized seed (MITWPUB1) with biosurfactant (10 mg); P3, bacterized seed (MITWPUB1) with biosurfactant (30 mg); P4, bacterized seed with biosurfactant (10 mg) and fungal suspension of *S. rolfsii*; P5, bacterized seed with the biosurfactant (30 mg) and fungal suspension of *S. rolfsii*.

### 3.7 Potential bacterial isolate identification and characterization

**FIGURE 6:**
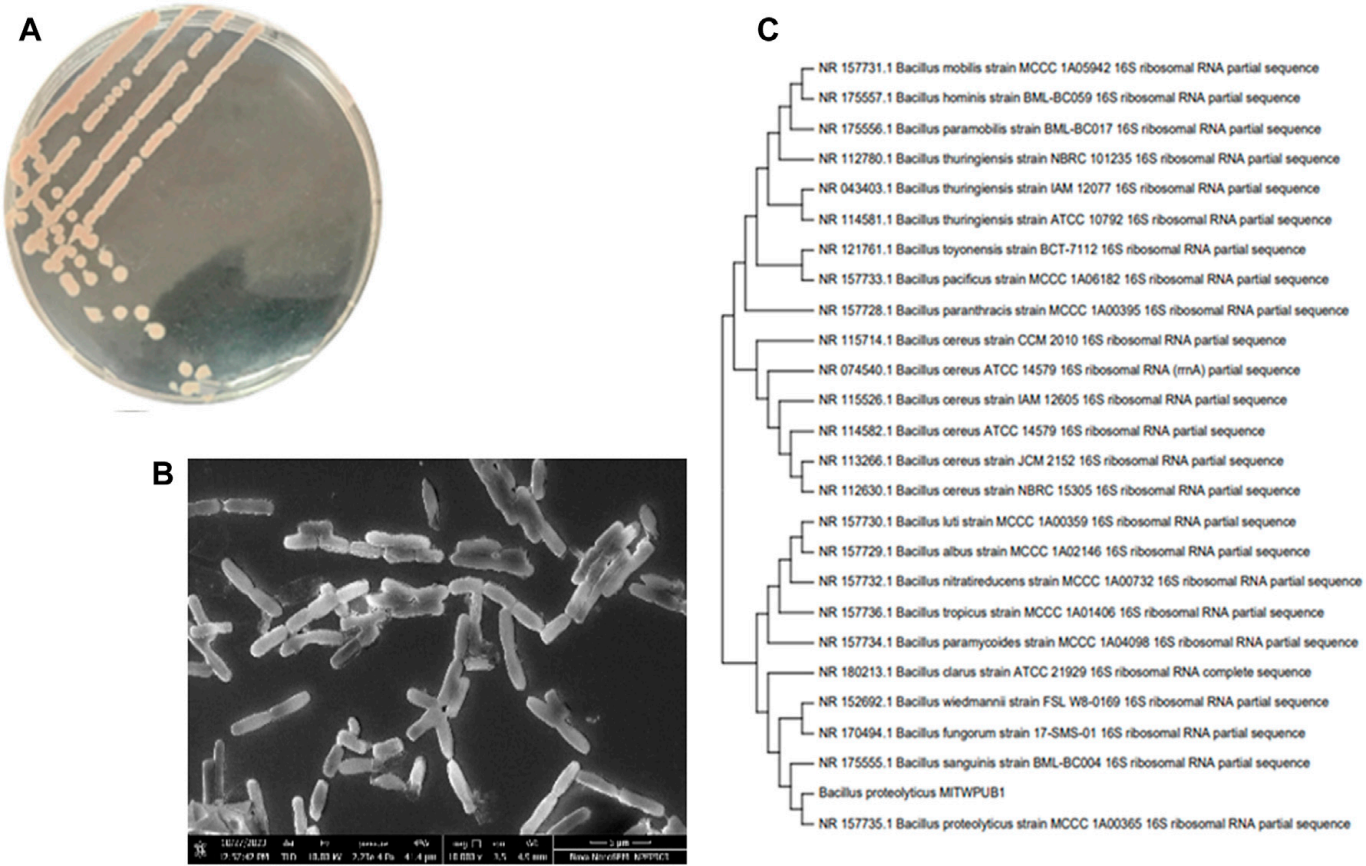
**(A)** Morphological traits of *B. proteolyticus* MITWPUB1 on LB agar plate incubated at 37°C for 24 h; **(B)** scanning electron microscopy of *B. proteolyticus* MITWPUB1 on LB agar plate incubated at 37°C for 48 h. **(C)** Phylogenetic tree constructed using MEGA version 11. The bacterial isolate showed 99.2% similarity with the members of the *Bacillus* genera.

The bacterial isolate colony was found to be round, viscoid, and slightly convex with a light pink pigmentation behavior (Table 3; Figure 6A). The microscopic characteristics revealed that the cells in the SEM images of the bacterial isolate were Gram-positive, non-motile, rod-shaped (Figure 6B), and formed endospores. The biochemical analysis of the isolate was positive for the catalase, indole, nitrate reductase, gelatinase, and oxidase tests (Table 4). The culture showed growth up to 4% NaCl with a pH range of 3–11. The optimum conditions were found to be 37°C at 0% NaCl and pH 7 (Supplementary Table S4). Blast analysis of the 16S rRNA of the MITWPUB1 sequences showed 99.2% similarity with the *Bacillus proteolyticus* strain MCCC 1A00365; hence, MITWPUB1 was identified as *B. proteolyticus* (Figure 6C), and its 16S rRNA sequences were deposited in the NCBI database with the accession number OQ726761.

**TABLE 3:** Microscopic and macroscopic characteristics of *B. proteolyticus* MITWPUB1.

Characteristic	Result
Macroscopic characteristics	
i. Size	Small or medium (2–3 mm)
ii. Color	White or light pink
iii. Margin	Entire
iv. Shape	Round (circular)
v. Elevation	Slightly raised
vi. Consistency	Viscid
vii. Opacity	Opaque
Microscopic characteristics	
i. Capsule	Absent
ii. Endospore	Present
iii. Motility	Non-motile
iv. Gram nature	Gram-positive

**TABLE 4:** Biochemical analysis of biosurfactant-producing isolate *B. proteolyticus* MITWPUB1.

Test	Result
Catalase test	+
Oxidase test	+
Indole test	+
Methyl red test	+
Voges–Proskauers test	-
Citrate utilization test	+
Hydrolysis of starch	+
Gelatin hydrolysis test	+
Nitrate reduction	+
Glucose	A
Sucrose	A
Lactose	A
Maltose	AG
Mannitol	AG
Sorbitol	AG

+, positive; -, negative; A, acid; AG, acid/gas.

## 4 Discussion

Hydrocarbons are some of the most prevalent environmental pollutants that are spilled by intense industrial activities. In such disturbed conditions, the growths of the macro and microorganisms are affected severely. Microbes are ubiquitous in nature and impact the environment in many positive and negative ways. These organisms oversee many activities, such as nutrient cycling, which impact soil quality, health, and nutrient enrichment. All these processes stimulate growth and productivity while reducing diseases in plants (Nabi, 2023). However, isolating organisms that possess the ability of hydrocarbon remediation requires a niche environment. Numerous bacteria, including *Pseudomonas, Bacillus, Streptomyces,* and *Stenotrophomonas* species, have been reported by Cai et al. (2015) from a hydrocarbon-contaminated site. Later, Shweta et al. (2021) reported isolation of beneficial PGPRs from oil-contaminated soil, but there are only limited reports available to date. In the present study, all 14 bacterial isolates of hydrocarbon-contaminated soil sites were found to have efficient PGP traits.

PGPB influences the growth of a plant directly and indirectly by maintaining soil equilibrium, safeguarding the health of humans and the environment (Pellegrini et al., 2023), and playing essential roles in maintaining sustainability. IAA is a vital plant hormone that promotes the number, size, weight, root hairs, and soil contact area of the lateral roots of a plant (Chandra et al., 2018), which in turn improves the colonization behavior of microorganisms and enhances the plant’s nutrient availability (Chamkhi et al., 2022). HCN is a significant metabolite that inhibits the growth of deleterious microbes and impacts plant growth and productivity. Tomaž and Aleš (2016) reported phosphorus availability by rhizobacteria and noted that the plant hosts were improved by HCN generation. Phosphorus is the second most crucial macronutrient required by a plant for its growth and development after nitrogen (Lebrazi et al., 2020). In this study, among the 14 bacterial isolates, *B. proteolyticus* MITWPUB1 was found to be the highest producer of IAA and HCN as well as a solubilizer of phosphorus, indicating its potential for use as a PGP bacterial isolate.

It is widely acknowledged that microbes rely on hydrocarbons or vegetable cooking oil as economical and renewable substrates for large-scale BS production, especially the organisms in the *Bacillus* sp*.* (Hisham et al., 2019); they use them as energy sources to produce BSs (Varjani and Gnansounou, 2017). The genus and species of the organism, as well as its culture conditions, significantly impact the yield of the BS (Pardhi et al., 2020; Pardhi et al., 2022). To identify the potential BSs, drop collapse and emulsification index were reported as the most important tests (Walter et al., 2010; Shah et al., 2016). According to research by Meena et al. (2021), microbes with emulsification activities of at least 65% or more are reportedly significant producers of BSs that can be used in multiple domains. In the present study, the drop collapse test was found to be positive for MITWPUB1 within 30 s, which highlights its efficient BS production ability. Moreover, MITWPUB1 was reported to have the highest emulsification index activity; therefore, MITWPUB1 was selected for all further assays for its efficient PGP traits and BS production. The BS produced by MITWPUB1 showed stable emulsification at various conditions of pH 7 and 9, temperatures 37°C and 50°C, and 4% salinity. The stability of emulsification highlights the ability of the bacterial isolate for use as a remediator at sites contaminated with xenobiotic compounds. Similar observations were reported for the BS from *Bacillus licheniformis* DS1 by Purwasena et al. (2019) at high temperature, varying pH, and increasing NaCl concentrations.

The BS showed spots for carbohydrate, lipid, peptide, and amino acid moieties. The Rf values for the sugars, peptides, amino acids, and lipids were found to be in accordance with the reports of Banerjee and Ghosh (2021) and Balan et al. (2016). According to Inès and Dhouha (2015), glycolipids are a form of BS made up of lipid and carbohydrate moieties. The inclusion of lipid, protein, amino acid, and peptide moieties emphasizes the presence of lipopeptides as a form of BS (Carolin et al., 2021; Zargar et al., 2022). It was discovered that the BS of MITWPUB1 contained a combination of the glycolipid and lipopeptide classes, so additional FTIR analyses of the BS were performed. The presence of the glycolipid BS was suggested by the peaks at 3,404, 2,925, 2,856, 1,635, and 1,439 cm^-1^ (Patowary et al., 2017), which is consistent with the findings of this investigation. In amine-containing groups, a wide absorbance peak between 3,450 cm^-1^ and 3,220 cm^-1^ is typical of N-H stretching vibrations, whereas the peaks close to 1225 cm^-1^ correspond to peptide ponds (Banerjee and Ghosh, 2021).

The results of LC-MS analysis also support the presence of amino acids, peptide linkages, and lipid moieties, indicating that a lipopeptide BS class was also produced by the bacterial isolate *B. proteolyticus* MITWPUB1. The *Bacillus* sp. is known to produce lipopeptides (Ndlovu et al., 2017) and a blend of BSs containing both lipopeptides and glycolipids (Fangyu et al., 2013). The sample showed the presence of different classes of phospholipids often grouped under the lipopeptide class of BSs (Gayathiri et al., 2022). Furthermore, both glycerolipids and sphingolipids were found in the sample. The presence of different phospholipids, glycerolipids, and sphingolipids as blends in the samples suggests possible accumulation of particulate BSs in extracellular vesicles owing to changes in the membrane composition of the *Bacillus* sp. under exposure to hydrocarbons (Vinayavekhin et al., 2015). Monteiro et al. (2007) reported that extracellular membrane vesicles partition hydrocarbons to form microemulsions that play essential roles in hydrocarbon assimilation and degradation. Considering *B. proteolyticus* MITWPUB1 as an isolate of hydrocarbon-contaminated soil, the addition of vegetable oil as a carbon source in the MSM at the time of BS extraction impacted the possibility of formation of particulate BSs by MITWPUB1. Interestingly, LC-MS analysis also showed the presence of lysophospholipids in the sample that can act as surfactants (Stafford and Dennis, 1987). These surface-active compounds are generated naturally in biological membranes by phospholipases. The presence of bacterial ceramides, a class of sphingolipids, in the sample also indicates the industrial potential of the bacterial BS (Akbari et al., 2018). In addition to the BS, the isolate produces polyamines, as found in the LC-MS analysis. The genes for polyamine synthesis were also reported earlier in the BS-producing *B. aquimaris* strain (Waghmode et al., 2017). Anwar et al. (2015) reported that ethylene levels can be altered by these metabolites to improve plant/root growth and development. LC-MS-based metabolite profiling also indicates the presence of IAA derivatives (Tang et al., 2023) and ACC (ethylene biosynthesis intermediate), indicating their valuable role in PGP (Gamalero et al., 2023).

Plants infected by pathogens can experience significant losses in agricultural production ranging from 10% to 40% (Savary et al., 2019). *S. rolfsii* is a resistant fungus that persists in soil through sclerotia; synthetic chemicals have typically been used for decades to control the disease, which have serious consequences in terms of enhancing the resistance of the pathogen, altering the ecosystem equilibrium, entering the food chain, and causing biomagnification–bioaccumulation in the food web (Kim et al., 2004). The growing demand for environmentally friendly solutions in terms of biodegradability has emphasized the quest for new biomolecules. BSs are an emerging class of biomolecules with multifunctional abilities owing to their amphipathic moieties. In the present study, the poison food technique exhibited dose-dependent antifungal effects of the BS against *S*. *rolfsii*; the dose-dependent trend noted in this study was also documented by Karamchandani et al. (2022). In the present study, the dry weight of the fungal biomass showed growth inhibition at two different BS concentrations. The highest inhibition of 72% was observed at the higher concentration of the BS. The BS decreases the effects of the harmful bacteria by generating an antibiofilm layer on various surfaces, which structurally destroys the intercellular net and conidiophores, along with delaying or preventing sporulation, thereby lowering the quantity of biomass generated (Gaikwad, 2023). Mycelial cells are said to be lysed from the action of the BS, which destabilizes them by intercalating with the phosphatidylcholine and phosphatidylethanolamine bilayers (Abbasi et al., 2012; 2013; Sharma et al., 2021). Any disruptions in fungal mycelia or their structural integrity may impact the ability to spread and can be the reason for their non-survivability as fungal mycelia perform a vital function in the asexual cycle that permit the organisms to colonize and live. The amphiphilic properties of BSs enable them to connect with plasma membranes, bind to pathogens, and release their internal contents, leading to cellular lysis and rupture (Crouzet et al., 2020; Kumar et al., 2021). The phospholipid bilayer of the cell membrane is disrupted by mycelial thinning, which can result in the loss of electrolytes, protein, and DNA (Borah et al., 2016; Monnier et al., 2019). In this study, mycelial thinning along with other structural deformations are clearly observed in the SEM images, supporting the impact of the BS in reducing the survivability of *S. rolfsii*.

The BS of the bacteria MITWPUB1 was tested to identify its toxicity in the plant *B*. *juncea* var local. The seeds coated with the BS had enhanced germination rate by 64% with respect to the control, indicating that the BS is not toxic to the plant. A higher concentration of the BS enhances the viability and colonization ability, which may be some of the major factors in enhancing seed germination and vigor index. This study shows that the BS can be utilized effectively to improve plant germination. Oluwaseun et al. (2017) reported similar findings for the BS isolated from *Pseudomonas aeruginosa* C1501 in promoting root elongation of plants; in their study, sawdust proved to be nontoxic to bacteria and successfully helped the organism maintain its viability. An increase in the CFU per mL highlights the nontoxicity of the carrier material (Kolet, 2014); this is a vital parameter that helps the organism to enhance plant growth development and production while maintaining the equilibrium status of the soil (Arora et al., 2014). In the BS-amended bioformulation, the CFU per mL was not reduced even after 240 h of incubation. The emulsifying capabilities of the surfactant, which provide desiccation protection, may be the reason for this optimum viability. Emulsification protects the bacteria against desiccation and death, enhancing the effectiveness of the biocontrol agent, according to extant studies (Laskowska and Kuczyńska-Wiśnik, 2020). Although the involvement of the BS in PGP and production are not extensively documented, the viability of the bacteria in the bioformulation determines the fate of the organisms in terms of their PGP features. Stringlis et al. (2018) reported that microbes producing BSs utilize them to enhance their motility, nutrient transport, competitive colonization, and microbe–host interactions. As less information is available in this regard, the topic is still open for investigation. Microbes colonize many different parts of the plants and show positive interactions that enhance plant growth and productivity. In this study, *B. proteolyticus* MITWPUB1 was found to effectively produce primary and secondary metabolites, such as IAA and HCN, while also aiding solubilization of phosphates. *B. proteolyticus* as a PGPR has been documented by a few researchers (Saran et al., 2020; Jasrotia et al., 2021; Li et al., 2023; Yang et al., 2023). Their results also indicate the potential for developing a bioinoculant-based bioformulation using *B. proteolyticus* MITWPUB1.

Plants growing in hydrocarbon- or pesticide-contaminated sites often have less growth and productivity. Hence, a metabolite that can remediate the concentration of pollutants is essential for the health of the plants, soil, and ecosystem. Reports by Awada et al. (2011) and Gayathiri et al. (2022) highlight the efficiency of BSs when used as emulsifiers to reduce hydrocarbon concentrations; they can also be used as adjuvants for pesticide formulations. Numerous investigations have identified the importance of BSs as chelating agents, with the potential to bind with trace elements to enhance the soil’s micronutrient bioavailability (Sachdev and Cameotra, 2013; Singh et al., 2018). Additionally, these BSs are excellent dispersing agents owing to their potent penetrating action, wetting, and amphiphilic qualities (Plaza and Achal, 2020). These qualities could greatly benefit bioformulations by assisting PGPB in colonizing plant roots while making phytohormones and other metabolites accessible to the plant. Mishra et al. (2020) reported the dynamic interplay of bacteria with PGP traits and BS production ability. As BSs are safe and biodegradable, it has been recommended that BS-based bioinoculants and bioformulations be implemented in the intricate rhizosphere environment (Santos et al., 2016).

It is possible to use BSs in three different forms: crude, refined, or combined with bacteria that produce them (Eras-Muñoz et al., 2022). In this study, the plant growth was improved and infection by *S. rolfsii* was reduced through the use of the crude BS and bacteria in a sawdust-based bioformulation. Using bacteria with PGP traits and BS production abilities offers a potential remedy by releasing essential biomolecules into the soil to maintain environmental sustainability. *B. proteolyticus* MITWPUB1 showed positive response in enhancing the seed germination rate. Yang et al. (2023) reported that the *B. proteolyticus* strain OSUB18 has the ability to increase induced systemic resistance in the *Arabidopsis* plant. Thus, *B. proteolyticus* MITWPUB1 that produces a BS to combat fungal phytopathogens can be used as a model for controlling their pathogenicity. Formulations based on PGP traits with BS may be used as biological replacements for chemical inputs in agricultural practices, with the additional advantages of increased performance and bacterial colonization in plant roots. Green surfactant-based products and formulations are required extensively in agriculture and agrochemical firms for significant contributions toward maintaining sustainability and green goals. The market for such products is increasing rapidly and is predicted to increase exponentially in the coming years (Hamid et al., 2021). Carbon dioxide emissions are a major cause of climate changes that consequently affect plant productivity and the environment drastically. Replacing synthetic or fossil-derived surfactants could reduce CO_2_ emissions by 8% and prevent the release of nearly 1.5 million tons of CO_2_ into the atmosphere (Silva et al., 2024). This extensive drop in CO_2_ can play a significant role in the SDG 13 climate action in the context of carbon neutrality (Miao et al., 2024), which can make the environment more sustainable in the future. Bioformulations containing BSs can be used as dispersants and carriers for PGP microorganisms, in which they work as emulsifiers or wetting and dispersion agents that have antibacterial, insecticidal, and antiviral effects (Arora and Mishra, 2016). This enhances the soil quality, aggregation, and structure to increase nutrient availability, improve soil remediation, promote microbial activity, facilitate plant–bacterial interactions, increase water retention, and foster plant growth and immunity (Khare and Arora, 2021; Kumar et al., 2021; Datta et al., 2023; Silva et al., 2024). These significant effects are a strong reason for transforming the manner in which food is produced and consumed globally and can play vital roles as gamechangers for accomplishing food security (SDG 2) (Arora and Mishra, 2023). Additionally, there is an urgent need to identify more potential nonpathogenic strains with PGP traits and BSs, which could herald a major paradigm shift (Mishra et al., 2020) to accomplish sustainability development goals. It is expected that the global market for BSs will increase at a compounded annual growth rate of 5.80% between 2024 and 2032, achieving profits of approximately USD 4.33 billion by 2032 (TechSci Research, 2023), which can be a significant factor in enhancing the capital gain. *B. proteolyticus* has antioxidant and probiotic properties (Li et al., 2019; Zeng et al., 2021; Sathiyaseelan et al., 2022) that allow its use as a model test organism against the proliferation of other phytopathogens to enhance plant productivity in a sustainable manner.

## 5 Conclusion

In this research, *B. proteolyticus* MITWPUB1 was shown to have the ability to produce BSs as well as an array of other PGP traits, demonstrating its value as a bioinoculant for increasing soil and plant production. Thus, this work highlights the potential for development of biostimulants that act as green alternatives to conventionally used chemical fertilizers and pesticides. According to the sustainability development goals, there is a vital requirement to implement green compounds to achieve and maintain sustainability in the environment. Despite the benefits of BSs, their applications in the agrochemical industries are constrained. The precise functions of surfactants as biocontrol facilitators are not adequately understood and require further research. It would be intriguing to investigate the possibility of rhizoremediation and phytoremediation using a potential BS-producing isolate with PGP traits, like *B. proteolyticus* MITWPUB1, on crops experiencing abiotic and biotic stresses. Consequently, to mitigate the harmful effects of synthetic surfactants, there should be more emphasis on green surfactants. However, more research is needed to identify BSs and their interactions with other plants, deleterious microbes, and other PGP metabolites to enhance the applicability of such multifunctional molecules.

## Data Availability

The original contributions presented in the study are publicly available, and the corresponding data can be found here: MetaboLights, accession number: MTBLS9314.
