# Supplementary material for: Bioformulation of Bacillus proteolyticus MITWPUB1 and its biosurfactant to control the growth of phytopathogen Sclerotium rolfsii for the crop Brassica juncea var local, as a sustainable approach
Source: Front Bioeng Biotechnol. 2024 Apr 19;12:1362679. doi: 10.3389/fbioe.2024.1362679 (PMC11066288; doi:10.3389/fbioe.2024.1362679)
Supplement: Supplementary file 1 [file DataSheet1.docx]

**Supplementary Table S1.** **Screening of the bacterial Isolates for the ability of the phosphate solubilization and production of Indole acetic acid.**

| Bacterial Isolates | PSB – Halo zone (mm) | IAA concentration (μg per mL) |
| --- | --- | --- |
| MITWPUB1 | 14.2 | 48 |
| MITWPUB2 | 12 | 36 |
| MITWPUB3 | 10 | 21 |
| MITWPUB4 | 9.8 | 22 |
| MITWPUB5 | 11 | 28 |
| MITWPUB6 | 7 | 12 |
| MITWPUB7 | 8 | 17 |
| MITWPUB8 | 4 | 16 |
| MITWPUB9 | 4 | 12 |
| MITWPUB10 | 7 | 16 |
| MITWPUB11 | 6 | 18 |
| MITWPUB12 | 6 | 29 |
| MITWPUB13 | 3 | 20 |
| MITWPUB14 | 4 | 21 |

**Supplementary Table S2**. **Plant growth promoting traits, drop collapsing activity and emulsification index of the bacterial isolates**.

| Bacterial Isolates | IAA Production | HCN Production | Phosphate Solubilization | Drop Collapsing activity | Emulsification Index (E_24_%) |
| --- | --- | --- | --- | --- | --- |
| MITWPUB1 | +++ | +++ | +++ | +++ | 83 |
| MITWPUB2 | +++ | ++ | +++ | ++ | 75 |
| MITWPUB3 | ++ | ++ | ++ | ++ | 72 |
| MITWPUB4 | ++ | +++ | ++ | +++ | 58 |
| MITWPUB5 | +++ | ++ | +++ | + | 8 |
| MITWPUB6 | + | ++ | ++ | +++ | 12 |
| MITWPUB7 | ++ | ++ | ++ | + | 4 |
| MITWPUB8 | ++ | + | + | + | 4 |
| MITWPUB9 | + | ++ | + | + | 12 |
| MITWPUB10 | ++ | ++ | ++ | +++ | 21 |
| MITWPUB11 | ++ | ++ | ++ | +++ | 24 |
| MITWPUB12 | +++ | ++ | ++ | ++ | 8 |
| MITWPUB13 | ++ | ++ | + | ++ | 4 |
| MITWPUB14 | ++ | + | + | +++ | 26 |

For IAA production: +++, 25 – 50 µg per mL; ++, 15 - 25 µg per mL; +, 0 – 15 µg per mL

For HCN Production : +++, Brown;++,Orange;+,Yellow

For Phosphate Solubilization : +++, 10 to 15 mm; ++,5 to 10 mm; +,1 to 5 mm

For Drop collapsing activity: +++,Within 1 min;++,After 1 min;+,Drop collapse after 5 min

**Supplementary Table S3. Liquid Chromatography–Mass Spectrometry (LCMS) analysis of the biosurfactant metabolite.**

| S. No | Formula | Compound ID | Name of the compound | Class of the Compound | RT | Mass |
| --- | --- | --- | --- | --- | --- | --- |
| 1 | C_43_ H_79_ O_13_ P | 3 | Phosphatidylinositol (34:2) | Phospholipids | 0.455 | 834.52 |
| 2 | C_45_ H_81_ O_13_ P | 4 | (2R)-1-[(Hydroxy{[(1S,2R,3R,4S,5S,6R)-2,3,4,5,6-pentahydroxycyclohexyl] oxy}phosphoryl)oxy]-3-[(9Z)-9-tetradecenoyloxy]-2-propanyl (13Z,16Z)-13,16-docosadienoate | Phospholipids | 0.458 | 860.54 |
| 3 | C_35_ H_66_ O_4_ | 19 | 1-O-(1Z-Tetradecenyl)-2-(9Z-octadecenoyl)-n-glycerol | Glycerolipids | 0.757 | 550.49 |
| 4 | C_41_ H_76_ N O_8_ P | 20 | 1-oleoyl-2-linoleyl-sn-glycero-3-phosphoethanolamine | Phospholipid | 0.761 | 741.53 |
| 5 | C_42_ H_80_ N O_10_ P | 21 | 1-stearoyl-2-oleoyl-sn-glycero-3-phosphoserine | Phospholipid | 0.761 | 789.55 |
| 6 | C_39_ H_74_ N O_8_ P | 22 | 1-Palmitoyl-2-linoleoyl PE | Phospholipid | 0.768 | 715.51 |
| 7 | C_40_ H_70_ N O_8_ P | 23 | (2R)-2-[(6Z,9Z,12Z,15Z)-6,9,12,15-Octadecatetraenoyloxy]-3-[(9Z)-9-tetradecenoyloxy] propyl 2-(trimethylammonio)ethyl phosphate | Phospholipid with a glycerol backbone | 0.818 | 723.48 |
| 8 | C_55_ H_96_ O_6_ | 52 | 1,3-Bis(palmitoyloxy)-2-propanyl (5Z,8Z,11Z,14Z,17Z)-5,8,11,14,17-icosapentaenoate | Fatty acid derivative | 1.656 | 852.72 |
| 9 | C_42_ H_83_ N O_3_ | 62 | Ceramide | Glycolipids | 1.661 | 649.63 |
| 10 | C_6_ H_13_ N O_2_ | 102 | Leucine | Amino acid | 2.467 | 131.0946 |
| 11 | C_8_ H_16_ N_2_ O_3_ | 117 | Gly-Leu | Dipeptide | 2.711 | 188.1159 |
| 12 | C_4_ H_~~7~~_ N O_2_ | 121 | 1-Aminocyclopropane-1-carboxylic acid | Amino acid | 3.123 | 101.0475 |
| 13 | C_23_ H_44_ N_6_ O_5_ | 125 | L-Prolyl-L-lysylnorleucyl-L-lysine | Peptide there | 4.353 | 484.3397 |
| 14 | C_28_ H_51_ N O_7_ P | 129 | Lysophosphatidylcholine(20:4/0:0) | Phospholipid | 4.417 | 544.3393 |
| 15 | C_40_ H_50_ O_4_ | 130 | 7,8-Didehydroastaxanthin | Carotenoids | 4.466 | 594.3735 |
| 16 | C_34_ H_52_ O_8_ | 132 | 2,16-Bis(2-methyl-2-butanyl)-6,7,9,10,12,13,20,21,23,24,26,27-dodecahydrodibenzo[b, n][1,4,7,10,13,16,19,22]octaoxacyclotetracosine | Fatty acid derivative | 4.471 | 588.3651 |
| 17 | C_51_ H_98_ N O_8_ P | 139 | (9Z,25R)-31-Amino-28-hydroxy-28-oxido-22-oxo-23,27,29-trioxa-28λ5-phosphahentriacont-9-en-25-yl (15Z)-15-tetracosenoate | Phospholipids | 4.615 | 883.7043 |
| 18 | C_5_ H_11_ N O_2_ | 157 | Valine | Amino acid | 5.465 | 117.079 |
| 19 | C_3_ H_10_ N O_4_ P | 159 | Ethanolamine | Phospholipid | 5.482 | 155.0348 |
| 20 | C_8_ H_16_ N_2_ O_3_ | 160 | N-acetyl lysine | Amino acid | 5.496 | 188.1151 |
| 21 | C_6_ H_13_ N O_2_ | 165 | N-alkyl glycine | Amino acid | 5.569 | 131.0944 |
| 22 | C_25_ H_53_ N O_7_ P | 167 | Lysophospholipid (17:0/0:0) | Phospholipid | 5.664 | 510.3547 |
| 23 | C_8_ H_18_ N_4_ O_2_ | 168 | L-Arginine Derivative | Carboxy ester | 5.724 | 202.1426 |
| 24 | C_11_ H_23_ N_3_ O_3_ | 169 | L-Lysine-L-Valine | Dipeptide- Amino acid | 5.764 | 245.1736 |
| 25 | C_14_ H_30_ N_4_ O_2_ | 178 | Acetylspermine | Polyamines | 5.79 | 286.2363 |
| 26 | C_6_ H_14_ N_2_ O_2_ | 182 | Lysine | Amino acid | 5.8 | 146.1056 |
| 27 | C_10_ H_23_ N_3_ O_2_ | 186 | L-lysine derivative | Amino acid | 5.84 | 217.1784 |
| 28 | C_9_ H_21_ N_3_ O | 193 | N8-Acetylspermidine | Polyamines | 5.922 | 187.1685 |
| 29 | C_16_ H_35_ N O_2_ | 217 | Hexadecasphinganine | Sphingolipids | 6.071 | 273.2665 |
| 30 | C_5_ H_15_ N O_4_ P | 219 | Phosphate of choline | Amines | 6.094 | 184.0733 |
| 31 | C_10_ H_23_ N_3_ O_3_ | 268 | Spermidine | Polyamine | 6.757 | 233.173 |
| 32 | C_16_ H_35_ N O_2_ | 288 | Hexadecasphinganine | Sphingolipids | 19.078 | 273.2669 |
| 33 | C_18_ H_36_ O_5_ | 289 | 9,10,18-trihydroxyoctadecanoic acid | Fatty acid ester | 19.148 | 332.2567 |
| 34 | C_29_ H_52_ O_2_ | 294 | 5-Tricosyl-1,3-benzenediol | Phenolic lipids | 22.487 | 432.3957 |
| 35 | C_43_ H_72_ O_5_ | 297 | Diacylglycerol (18:1n9/22:5n6) | Glycerolipid | 24.724 | 668.5401 |
| 36 | C_16_ H_33_ N O | 302 | Fatty amide | Lipid | 24.822 | 255.2563 |
| 37 | C_20_ H_22_ O_5_ | 305 | Oxydipropyl dibenzoate | Ester derivative | 24.98 | 342.1469 |
| 38 | C_20_ H_34_ O | 316 | Arachidonyl alcohol | Fatty alcohol | 26.828 | 290.2606 |
| 39 | C_21_ H_30_ N_4_ O_4_ | 320 | 1H-Indole-1-acetic acid, 2-[4-[(3-butoxypropyl)amino]-1,4-dioxobutyl]hydrazide | IAA derivative | 27.426 | 402.2265 |
| 40 | C_22_ H_36_ O | 338 | (4E,8E,12E)-4,9,13,17-Tetramethyl-4,8,12,16-octadecatetraenal | Lipid | 28.461 | 316.2769 |
| 41 | C_22_ H_39_ N O | 339 | (2E,4E,12Z)-N-(2-Methylpropyl)-2,4,12-octadecatrienamide | Lipid | 28.465 | 333.3033 |
| 42 | C_20_ H_41_ N O_2_ | 341 | N-acylethanolamines | Ethanolamide to octadecanoic acid | 28.882 | 327.3135 |

**SUPPLEMENTARY Table S4. Optimum conditions for the growth of bacteria B*acillus proteolyticus* MITWPUB1.**

| Optimum Conditions | Optical Density at 600 nm after 24 hours of Incubation |
| --- | --- |
| pH | |
| 3 | 0.159 |
| 5 | 0.425 |
| 7 | 0.875 |
| 9 | 0.631 |
| 11 | 0.699 |
| Temperature (Degree Celsius) | |
| 4 | 0.043 |
| 27 | 0.793 |
| 37 | 0.995 |
| 50 | 0.233 |
| NaCl | |
| 0 % | 0.956 |
| 3 % | 0.786 |
| 4 % | 0.844 |

**SUPPLEMENTARY FIGURE S1**. Standard graph of Indole acetic acid (IAA)


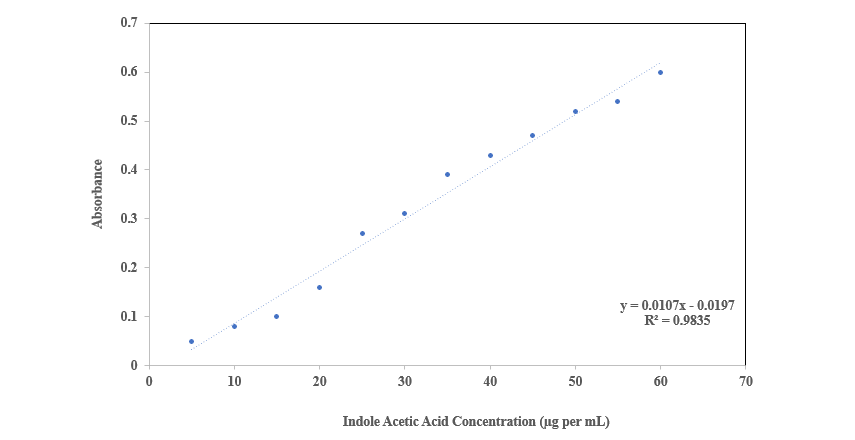


**SUPPLEMENTARY FIGURE S2.** Thin layer chromatograph of the biosurfactant of *B. proteolyticus* MITWPUB1. TLC Characterization of Biosurfactant: Left panel: Crude biosurfactant stained with iodine **(A)** Positive control, glucose; **(B)** Crude biosurfactant; **(C)** Negative control, glycine; Middle panel: Crude biosurfactant stained with ninhydrin; **(D)** Negative control, glycine; (E) Crude biosurfactant; **(F)** Positive control, glucose; Right Panel; **(G)** Crude biosurfactant stained with anthrone reagent.


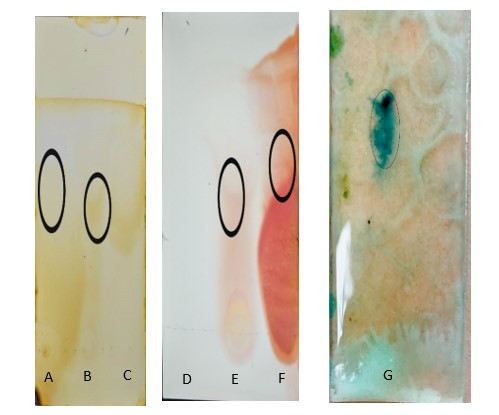


**SUPPLEMENTARY FIGURE S3.** Mass Spectrum Analysis of the peaks


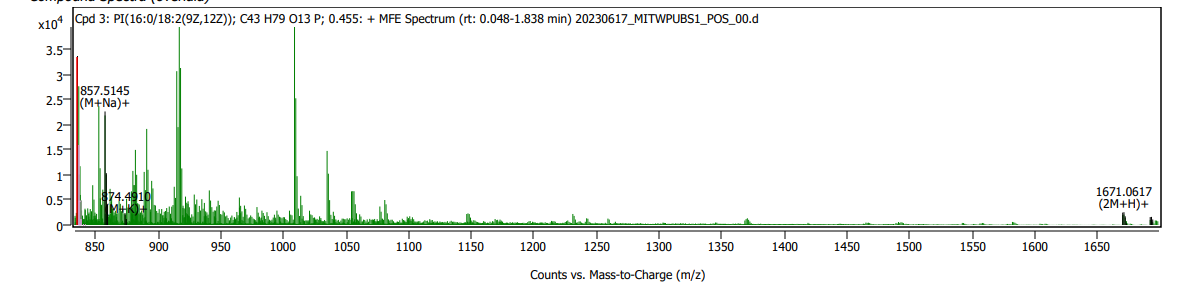


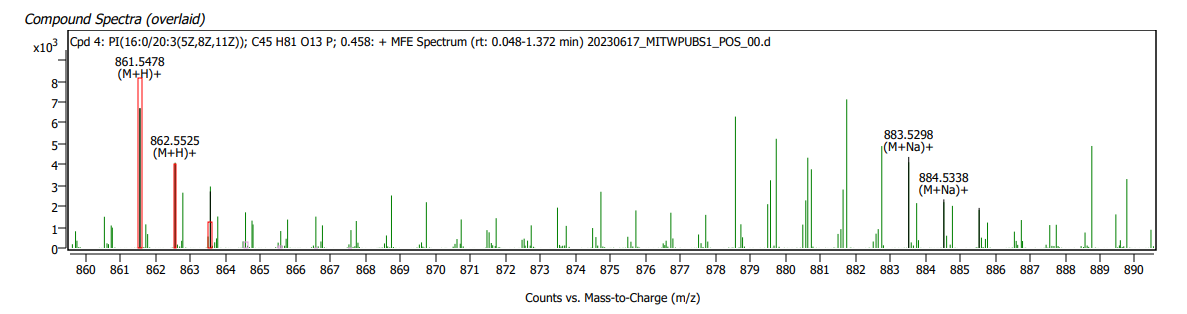


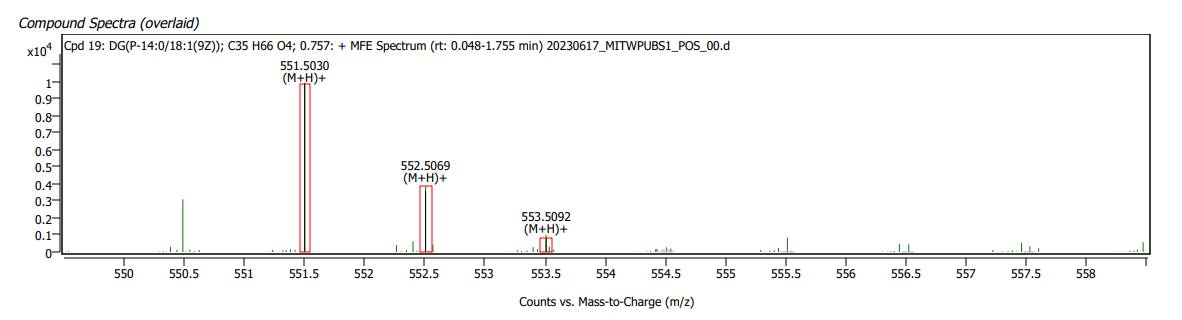


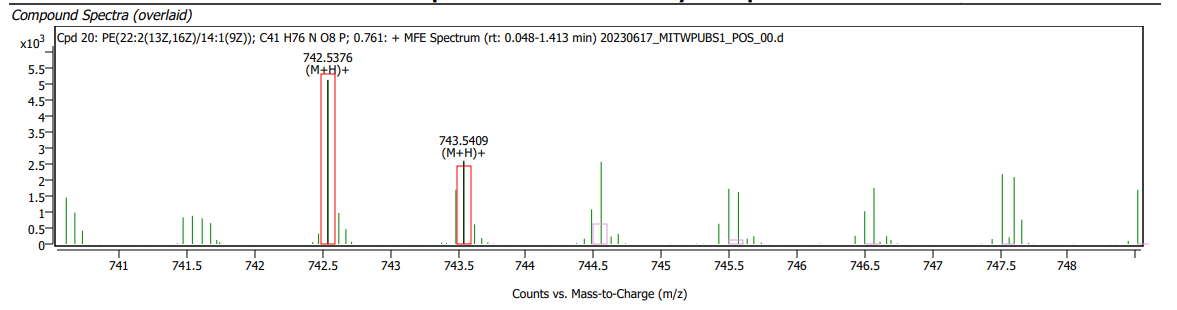


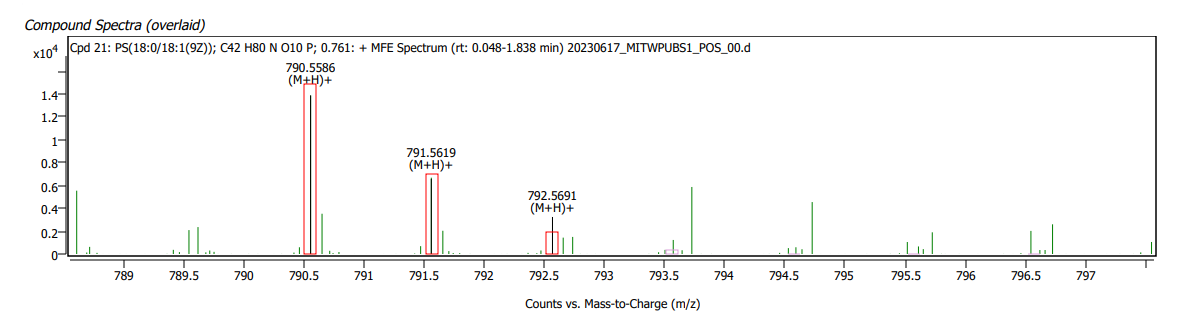


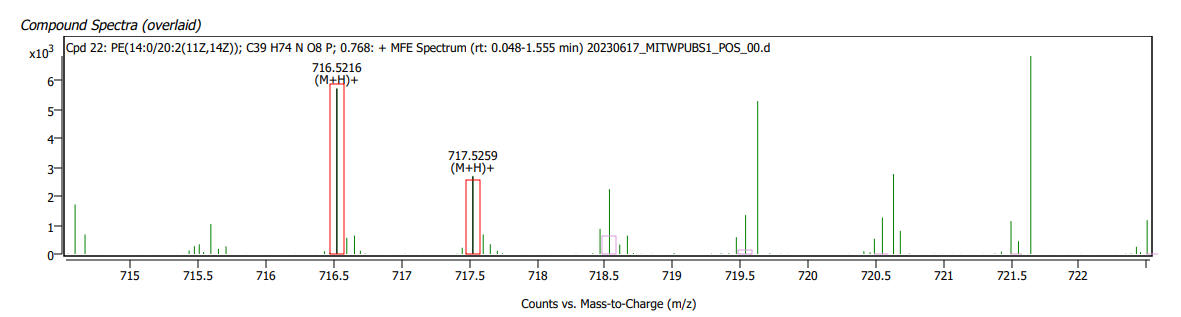


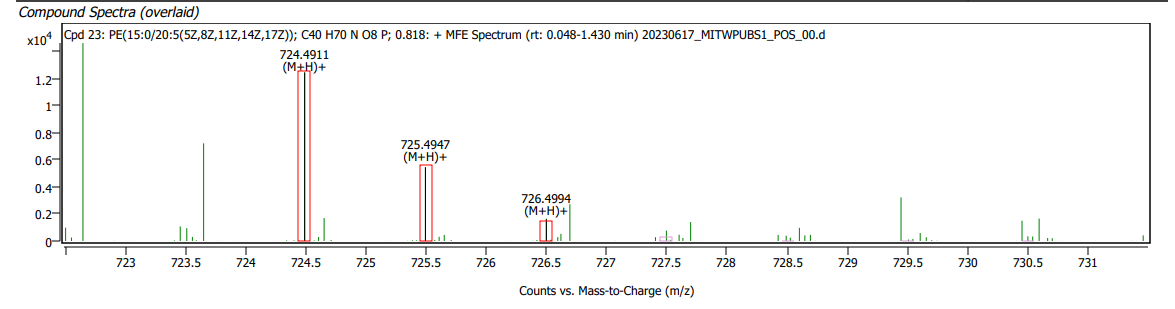


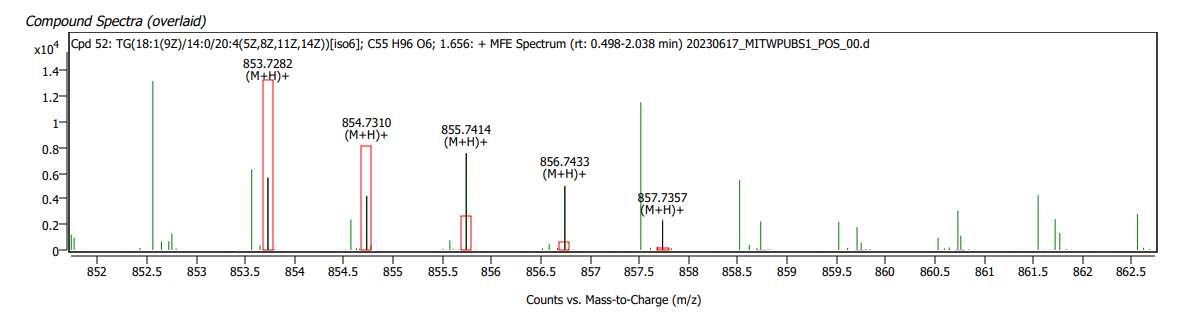


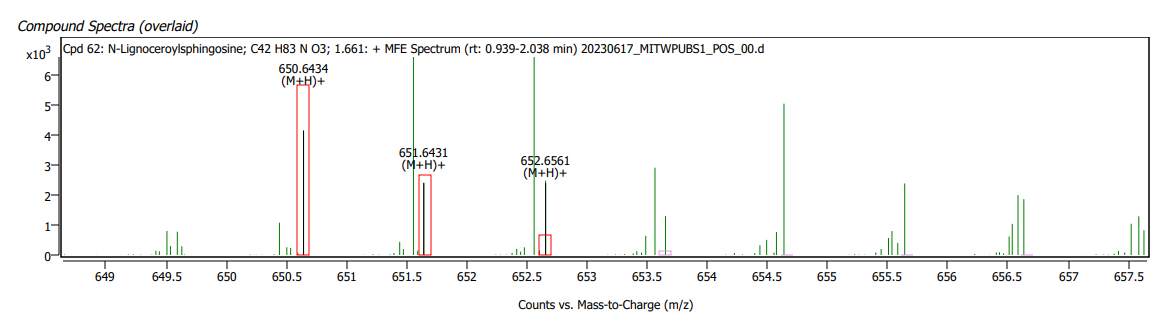


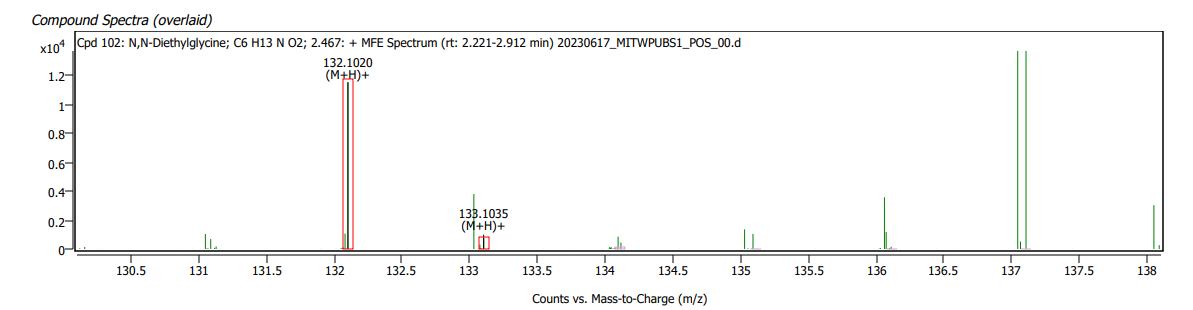


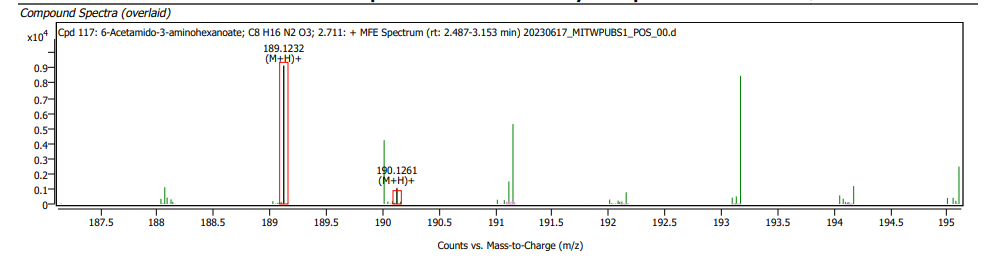


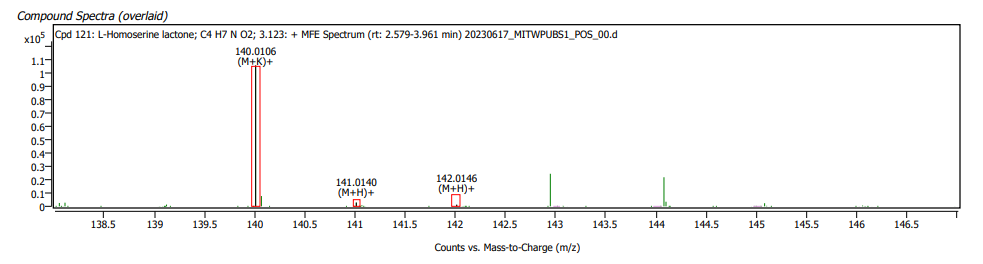


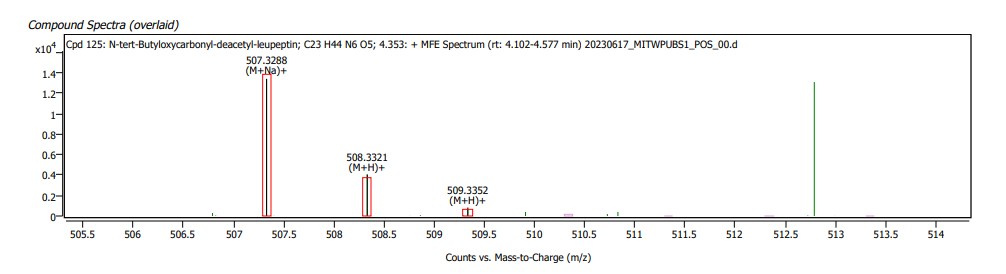


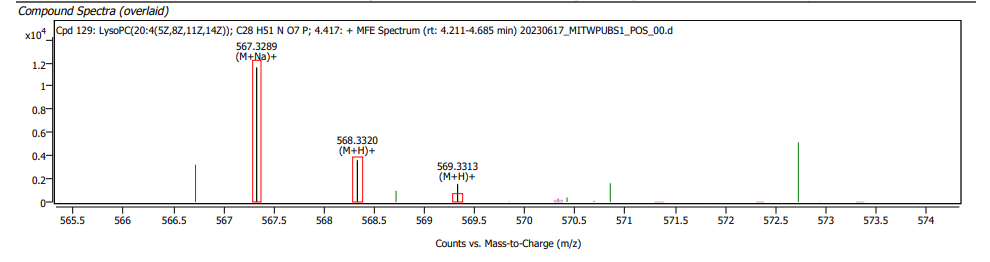


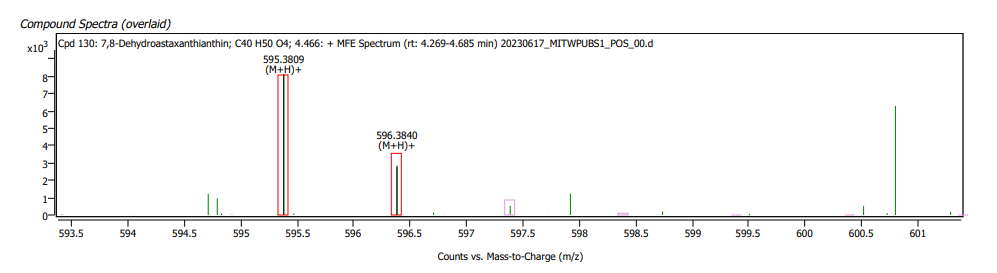


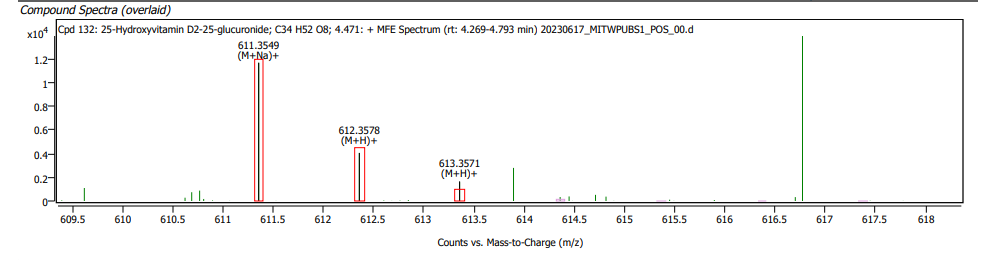


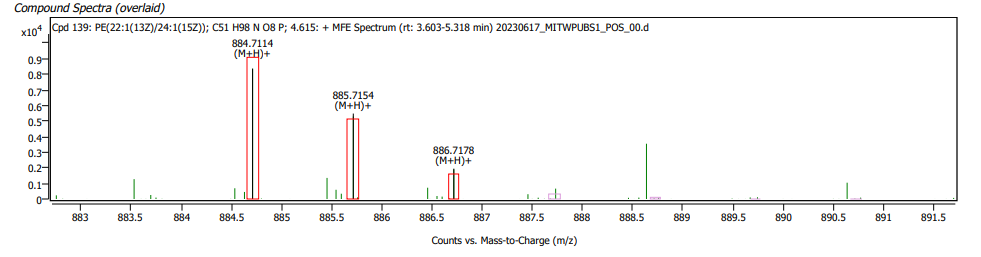


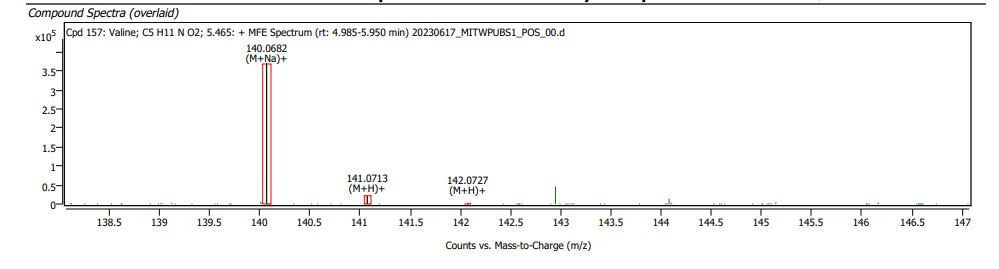


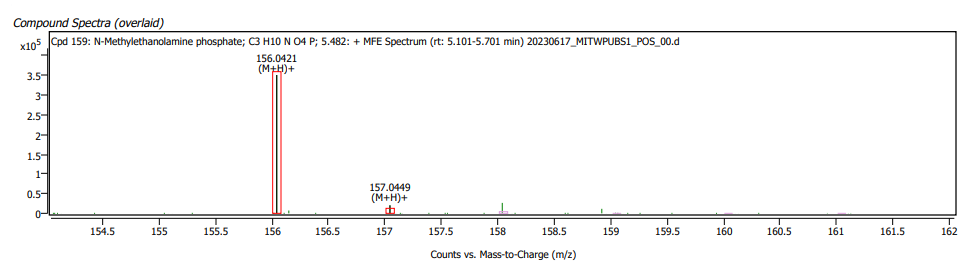


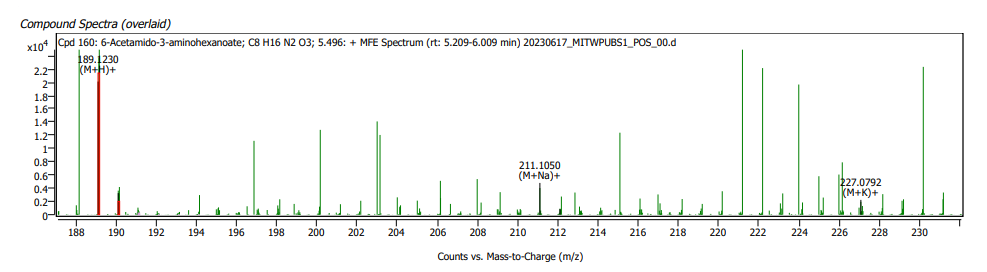


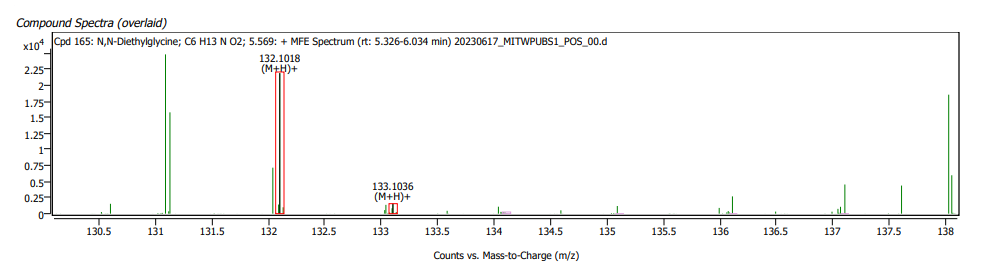


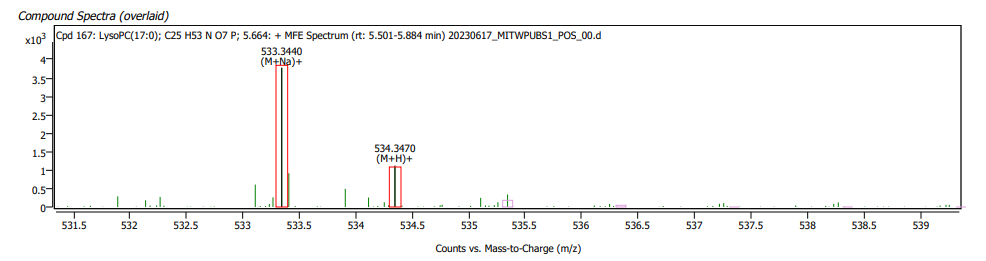


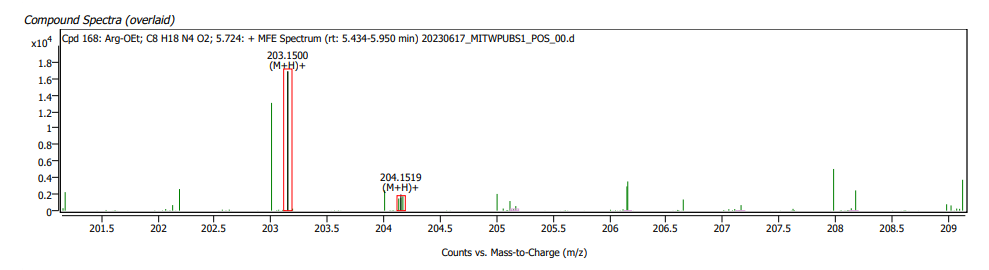


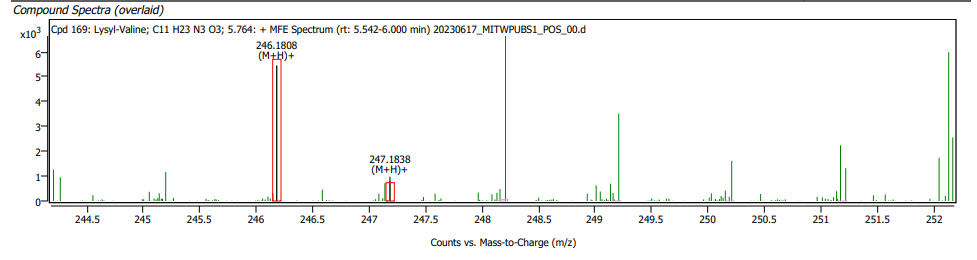


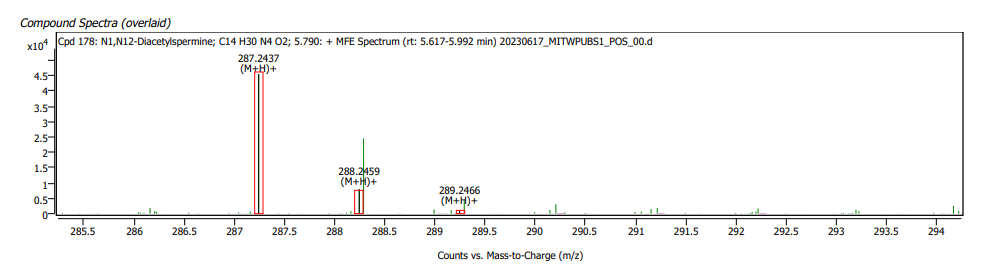


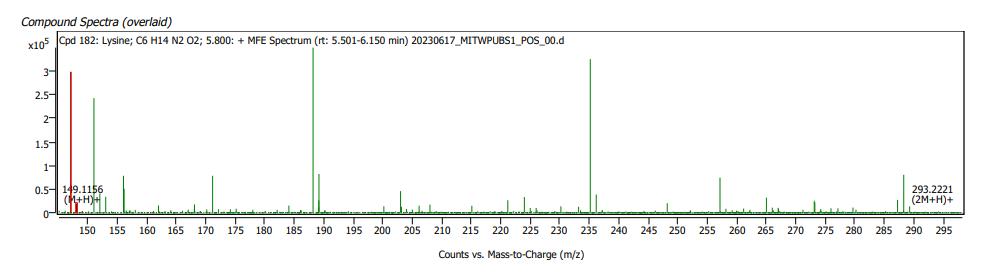


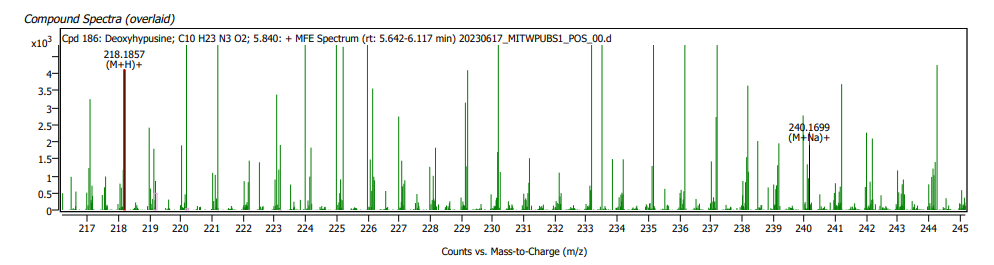


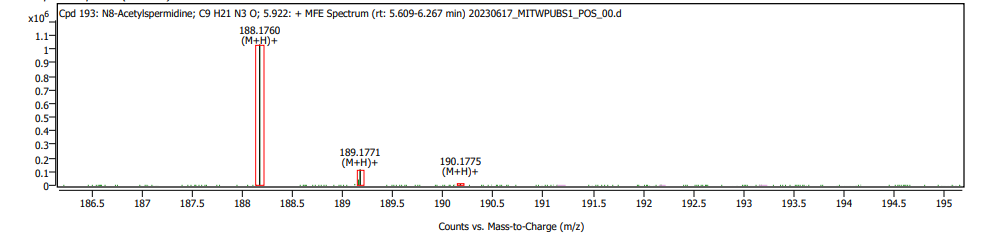


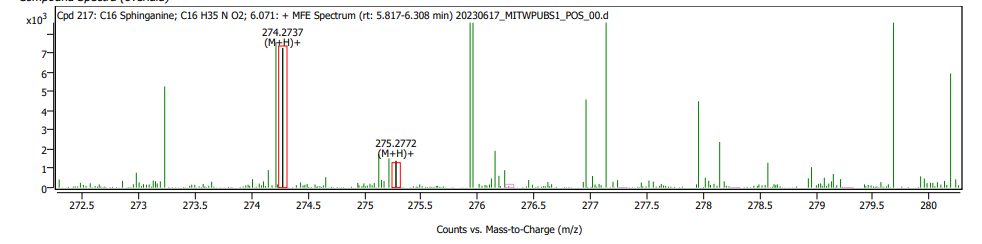


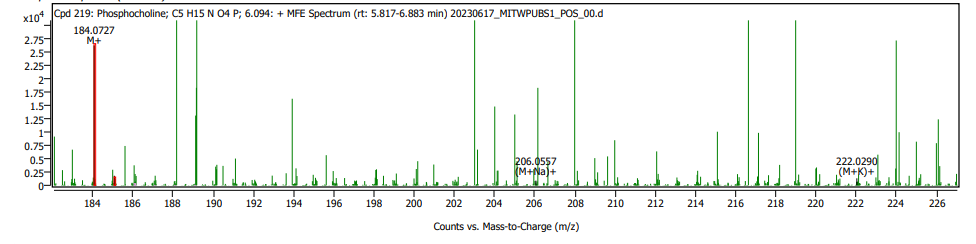


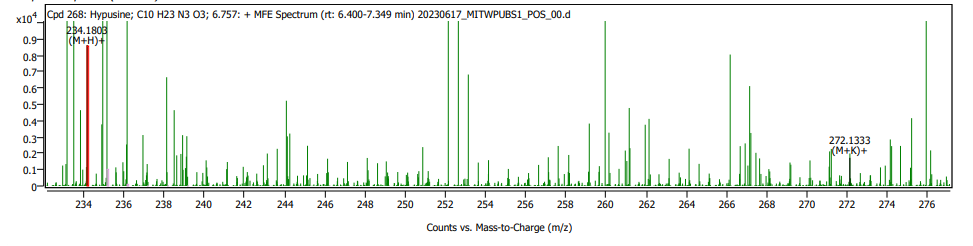


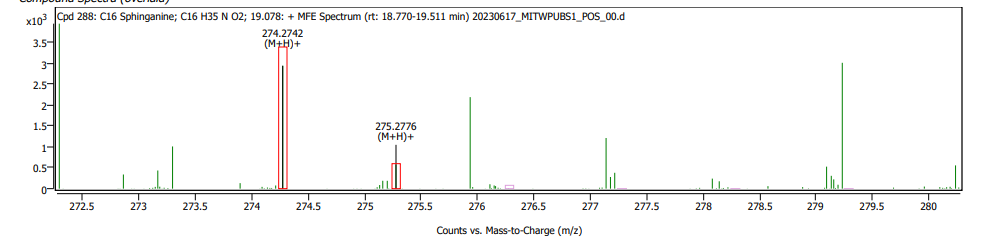


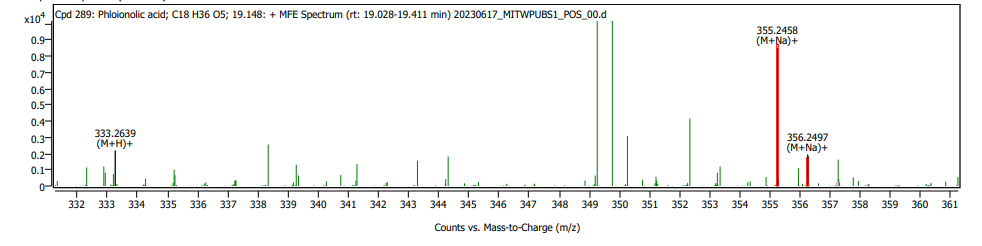


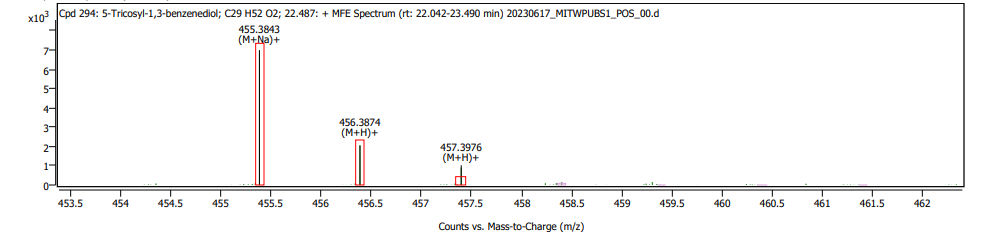


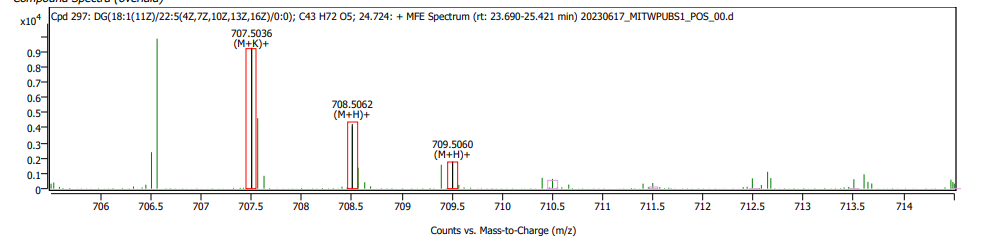


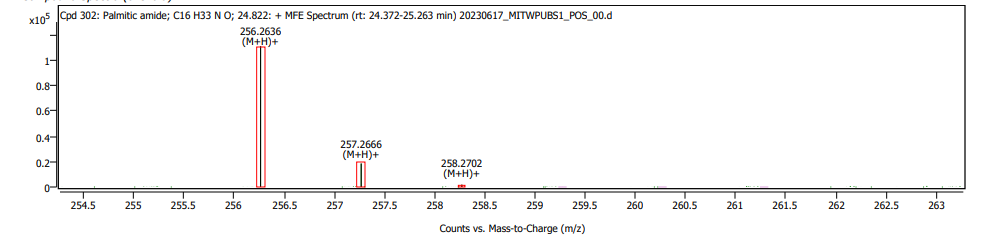


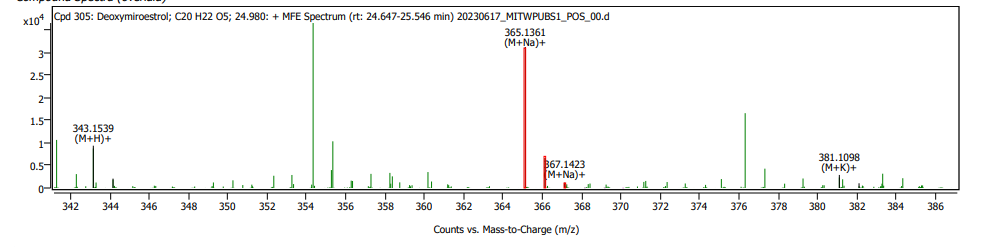


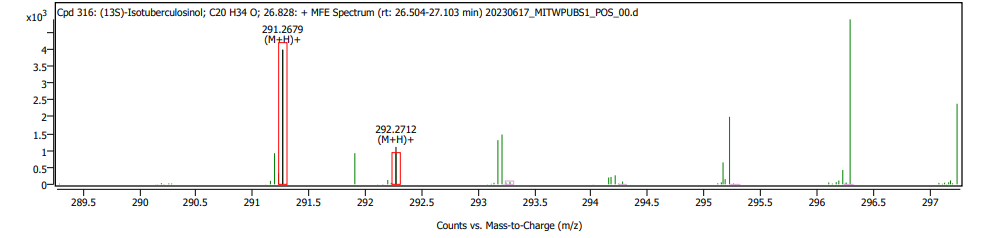


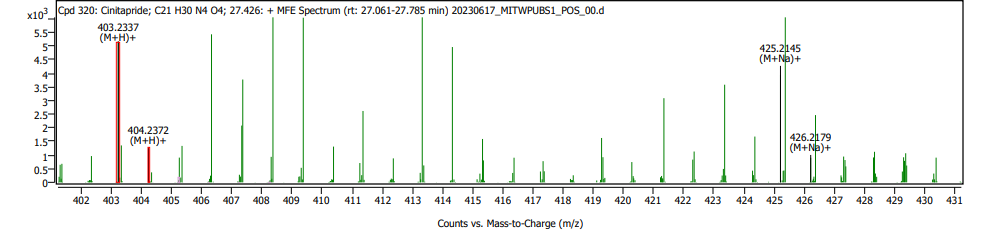


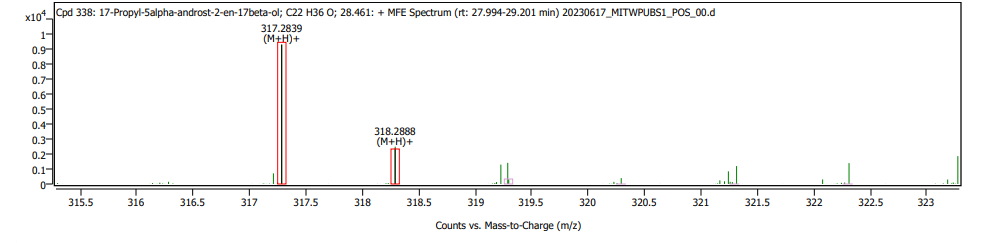


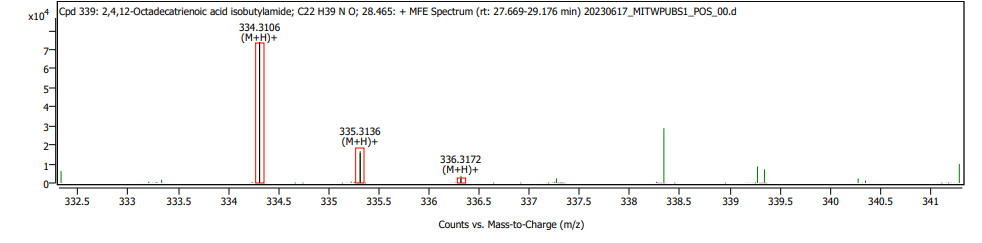


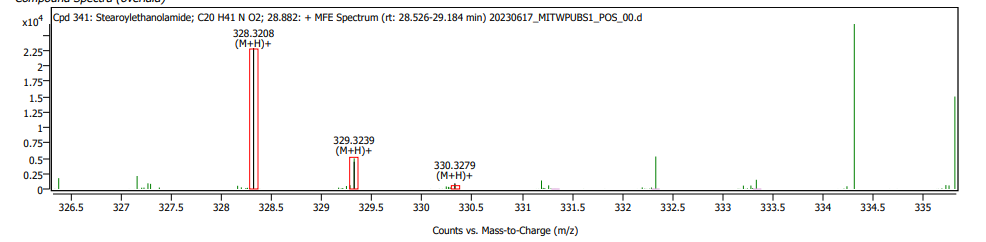


**SUPPLEMENTARY FIGURE S4.** Population density, colony forming unit; CFU per mL of the bacterial isolate MITWPUB1.All values are mean ± SD. Values in the bars followed by same letters indicate no significant difference (p ≤ 0.05) by Duncan’s multiple range test. Separately for 240 hours and 360 hours. T1, Control (Bacteria); T2, Sawdust + Bacteria; T3, Sawdust + Bacteria + Biosurfactant (30 mg per mL) after 240 and 360 hours of study.
